# Developmental coordination disorder in children – experimental work and data annotation

**DOI:** 10.1093/gigascience/gix002

**Published:** 2017-02-24

**Authors:** Lukáš Vařeka, Petr Brůha, Roman Mouček, Pavel Mautner, Ladislav Čepička, Irena Holečková

**Affiliations:** 1 University of West Bohemia, Univerzitni 8, 306 14, Plzen, Czech Republic; 2 University Hospital Plzen, Alej Svobody 80, 304 60, Plzen, Czech Republic

**Keywords:** developmental coordination disorder, event-related potentials, visual and audio stimulation, electroencephalography, reaction time

## Abstract

**Background:**

Developmental coordination disorder (DCD) is described as a motor skill disorder characterized by a marked impairment in the development of motor coordination abilities that significantly interferes with performance of daily activities and/or academic achievement. Since some electrophysiological studies suggest differences between children with/without motor development problems, we prepared an experimental protocol and performed electrophysiological experiments with the aim of making a step toward a possible diagnosis of this disorder using the event-related potentials (ERP) technique. The second aim is to properly annotate the obtained raw data with relevant metadata and promote their long-term sustainability.

**Results:**

The data from 32 school children (16 with possible DCD and 16 in the control group) were collected. Each dataset contains raw electroencephalography (EEG) data in the BrainVision format and provides sufficient metadata (such as age, gender, results of the motor test, and hearing thresholds) to allow other researchers to perform analysis. For each experiment, the percentage of ERP trials damaged by blinking artifacts was estimated. Furthermore, ERP trials were averaged across different participants and conditions, and the resulting plots are included in the manuscript. This should help researchers to estimate the usability of individual datasets for analysis.

**Conclusions:**

The aim of the whole project is to find out if it is possible to make any conclusions about DCD from EEG data obtained. For the purpose of further analysis, the data were collected and annotated respecting the current outcomes of the International Neuroinformatics Coordinating Facility Program on Standards for Data Sharing, the Task Force on Electrophysiology, and the group developing the Ontology for Experimental Neurophysiology. The data with metadata are stored in the EEG/ERP Portal.

## Data description

### Theoretical background and purpose of the study

The degree of motor development is usually assessed through clinical tests such as the Movement Assessment Battery for Children (MABC-2) [1]. There is an open question as to whether this disorder can be also diagnosed using other techniques, such as electroencephalography (EEG) or event-related potentials (ERPs). EPRs were primarily used as an alternative to measurements of the speed and accuracy of motor responses in paradigms with discrete stimuli and responses [2], and their general advantages when compared to behavioral measures seem to be worth investigating also in this case. There are two main advantages of the ERP technique over behavioral measures. An online measure of stimuli processing can be provided even when there is no behavioral response. The second advantage is that it can provide a continuous measure of processing between a stimulus and a response, making it possible to determine which stage or stages of processing are affected by a specific experimental manipulation [2].

Different studies have been published that investigate the link between EEG and DCD. For example, in de Castelnau et al. (2008), the authors suggest that spectral coherence of certain brain rhythms between different brain regions occurs in children with DCD [3]. It has been demonstrated that children with DCD have a limited ability to distinguish size, angles, area, and shape compared to children with normal development. Visuospatial processing disorders can be studied using the ERP-based protocol. Furthermore, the high comorbidity [4] between attention deficit hyperactivity disorder (ADHD) and DCD suggests the possibility of a common developmental anomaly of both disorders. Studies of ERP confirmed an attention deficit for both visual and auditory stimuli in children with ADHD [5,6]. Therefore, given the expected common anomaly in ADHD and DCD, children with DCD should have not only visuospatial attention deficit but also an auditory attention disorder [4]. Our objective was to design and perform event-related potential experiments that can potentially benefit from general advantages of this technique in comparison with traditional behavioral techniques for DCD diagnosis. Although traditional behavioral techniques are fast and relatively inexpensive, EEG, for example, does not need to rely on physical exercise itself and can be used if exercise is currently not possible for medical reasons. Furthermore, EEG can contribute to our understanding of the causes of DCD and potential comorbidities. In the long term, we would like to influence EEG through some special training (e.g., neurofeedback) and observe if such training can also influence severity of DCD.

### Participants

The tested subjects were 32 children of younger school age (21 males, 11 females, aged 7–10 years) from a primary school for children with impaired hearing in Pilsen. They were preliminary divided into three groups based on the level of their developmental coordination disorder, identified by the MABC-2 motor test [1]. The test evaluates motor performance on three main components: manual dexterity, aiming and catching, and balance. The decision was based on the total test score (also referred to as “sum SS”) according to a simple traffic light system that was proposed in Henderson et al. (2007)[1]. Children with score above 67 were in the green zone (no movement difficulty detected). The children who scored between 57 and 67 (inclusive) were in the yellow zone (at risk of having a movement difficulty). Finally, scores ≤56 denoted significant movement difficulty. However, because of a relatively small number of children in the yellow zone, for the purposes of further validation, we decided to merge the yellow zone and the red zone to achieve a group of children with or at risk of DCD. In summary, using the motor test, 16 children were at risk of or suffering from DCD (four of them were previously in the yellow zone), and 16 were without movement difficulties. All children were right-handed, and four children had corrected myopia. Most children suffered from hearing impairment. The level of hearing impairment was assessed using a hearing threshold test. The informed consent was signed by their legal guardians. All participants with some of the important metadata are listed in Table 1.

**Table 1:** List of all measured participants.

				Myopia	HT (db/1kHz)		MABC-2			Eye-blinks
ID	Sex	Age	Comorbidities	(MWG)	left	right	TS	SS	P	(%)
276	F	8y 7m	no	no	−5	5	77	9	37	50.4
277	F	7y 6m	ADD	no	−5	5	72	8	25	27.8
278	F	9y 1m	MBD	no	0	0	55	5	5	37
280	F	10y 0m	ADD	no	5	5	55	5	5	44.8
281	M	8y 4m	no	no	20	20	74	9	37	37.5
282	F	9y 11m	MBD	yes (MWG)	25	25	73	9	37	43.3
283	M	8y 4m	ADHD	yes	0	−5	54	5	5	57.5
284	M	8y 1m	AS	no	5	5	61	6	9	40.7
285	M	9y 0m	no	no	20	20	65	7	16	58
286	M	8y 10m	no	no	15	15	88	12	75	38.9
287	M	10y 0m	ADHD	no	5	20	54	5	5	18.2
289	M	8y 3m	DG, DO, DP	no	5	5	43	3	1	43
290	M	8y 7m	DL	yes (MWG)	10	0	54	5	5	29.4
291	M	8y 0m	DP	yes	5	10	85	11	63	37
292	M	7y 5m	DP	no	20	25	70	8	25	25.4
293	F	7y 0m	no	no	20	20	39	3	1	0
294	M	7y 2m	DP	no	5	0	73	9	37	31
295	M	7y 11m	ADD, DP	no	20	20	59	6	9	26.7
296	M	7y 7m	ADHD	no	0	−5	47	4	2	14
795	M	9y 11m	no	no	5	5	77	9	37	33.2
796	M	9y 6m	DLA	no	5	10	56	5	5	66
797	M	9y 9m	no	no	5	0	42	3	1	42.5
798	M	7y 2m	no	no	15	0	80	10	50	40.7
799	M	8y 1m	no	no	5	0	54	5	5	62.9
800	F	7y 7m	no	no	5	5	68	8	25	65.4
801	M	8y 9m	no	no	0	0	63	7	16	57.6
802	F	7y 9m	no	no	20	25	49	2	4	53.5
803	M	7y 3m	ADHD	no	15	5	71	8	25	67.5
804	M	9y 2m	no	no	5	0	93	14	91	67.7
805	F	7y 4m	no	no	20	20	75	9	37	60.9
806	F	8y 1m	no	no	10	10	85	11	63	39.6
807	F	8y 3m	no	no	5	5	97	15	95	47

Some of the most important metadata are included. The information about comorbidities was obtained from reports of educational and psychological counseling centers. AS, Asperger syndrome; DG, dysgraphia; DL, dyslexia; DLA, dyslalia; DO, dysorthography; DP, dysphasia; HT, hearing threshold; MBD, minimal brain dysfunction; MWG, measured without glasses; P, percentile; TS, total score; VI, visual impairment.

### Experimental procedure

**Figure 1:**
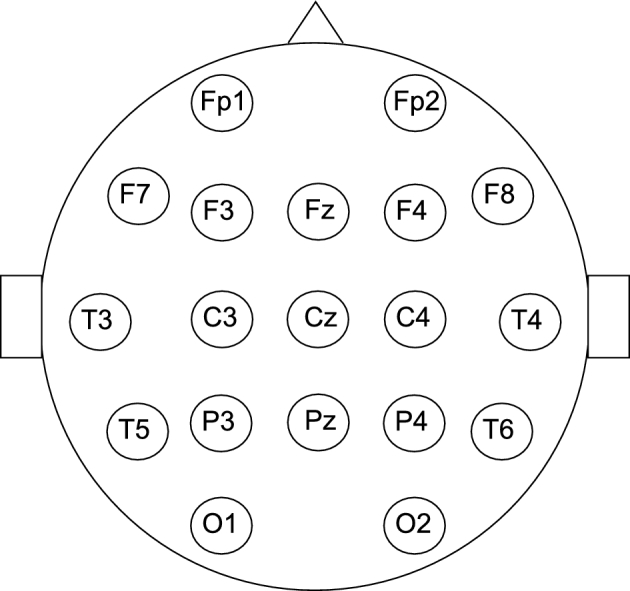
The locations of the electrodes attached in the 10-20 system.

**Figure 2:**
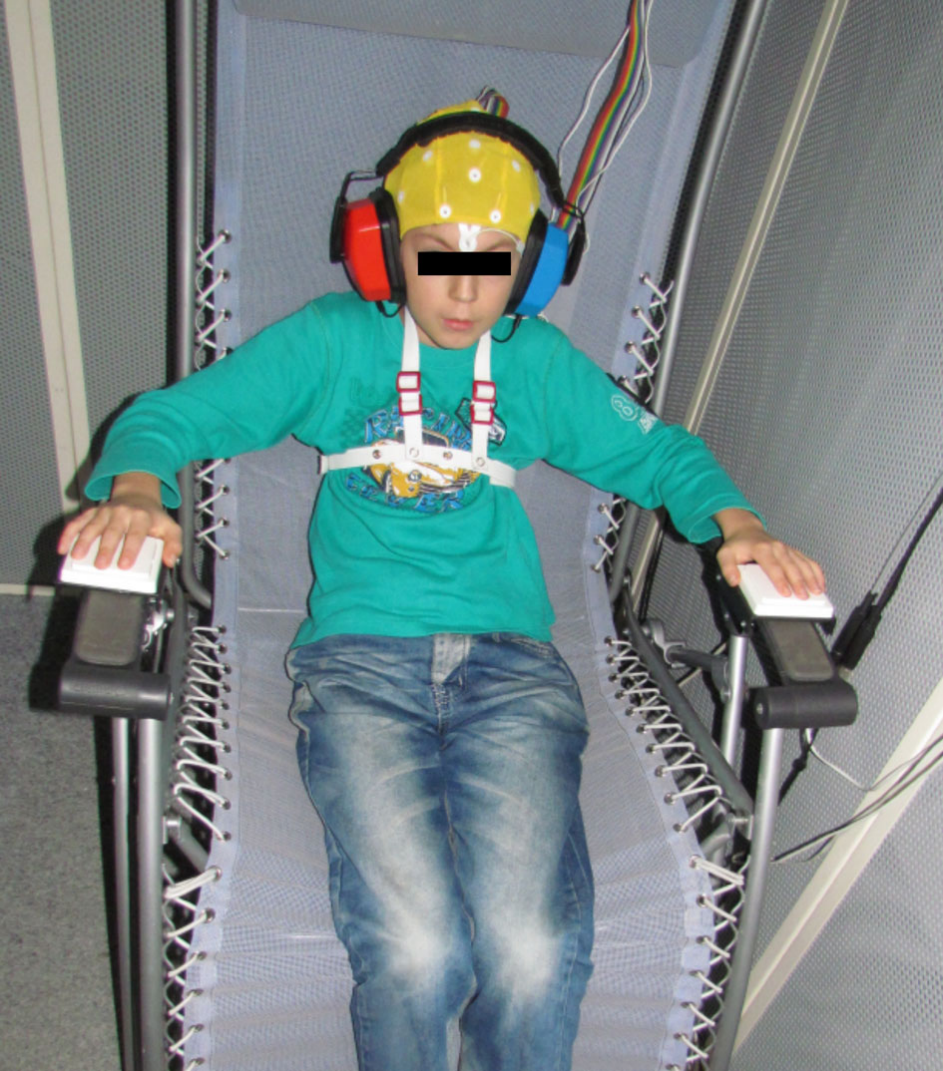
A participant during the experiment.

The following experimental procedure was applied:

- Each participant was acquainted with the course of the experiment and answered questions concerning his/her health.
- Each participant was given the headphones. The participant was taken to a soundproof and electrically shielded cabin. The hearing threshold for each ear was evaluated. The volume of auditory stimulation was calculated as follows: for each ear, the volume was set to be 50 dB higher than the hearing threshold. However, the volume never exceeded 75 dB.
- Each participant was given a standard 10–20 system EEG cap and headphones. Nineteen electrodes were used, as depicted in Fig. 1. The participant was taken to a soundproof and electrically shielded cabin; the reference electrode was placed at the root of his/her nose.
- The participant was told to watch the pictures on the screen, to listen to the sounds, and to respond to stimuli, as described in the “stimulation protocol” section.
- The cabin was closed, and both the data recording and stimulation started. Fig. 2 shows a participant during the experiment.
- After the experiment finished, the recorded data and collected metadata were uploaded to the EEG/ERP Portal [7].

### EEG data recording

#### Recording hardware

The standard 10–20 system EEG cap made by Electro-Cap International was used, for the experiment. The EEG cap contained 19 electrodes. The BrainAmp DC amplifier was used, with the sampling frequency set to 1 kHz. The raw signal was filtered using an analogue band-pass filter with the cut-off frequencies of 0.1 and 250 Hz. There were two buttons placed at the armrests of the chair for measuring the reactions of the participants (also depicted in Fig. 2).

#### Recording software

The BrainVision Recorder 1.2 [8] was used for recording and storing the EEG/ERP data in the BrainVision format. The impedance threshold was set to 10 *k*Ω; the real impedances for each experiment were stored in vhdr files. Presentation, version 16.3 (Neurobehavioral Systems), was used for stimulation [9].

#### Environment

All experiments were performed in a sound- and electrically shielded booth placed in an electrophysiology lab. EEG/ERP activity was recorded using the standard 10–20 international system, with the reference electrode placed at the root of the nose.

#### Stimulation protocol

The experimental protocol was based on multimodal stimulation, i.e., a combination of auditory and visual stimulation. The visual stimuli were represented by pictures of animals. The corresponding auditory stimuli were represented by sounds of the animals that occurred in synchronization with the visual stimuli. One of the pictures (a goat), occurring with a probability of 70%, was always associated with the correct sound and was the standard (non-target) stimulus. In rare stimuli, the sounds might be incorrectly associated with the animals. The rare stimuli included a barking dog (15%), meowing cat (5%), meowing dog (5%), and barking cat (5%). A total of 600 stimuli were used during the experimental session. Each experimental session was divided into two experimental runs, each containing 300 stimuli. During the experimental session, participants were asked to reply to each target stimulus (dog or cat sound) by pressing one button for sounds of a barking dog or meowing cat and the other button for sounds of a barking cat or meowing dog.

**Figure 3:**
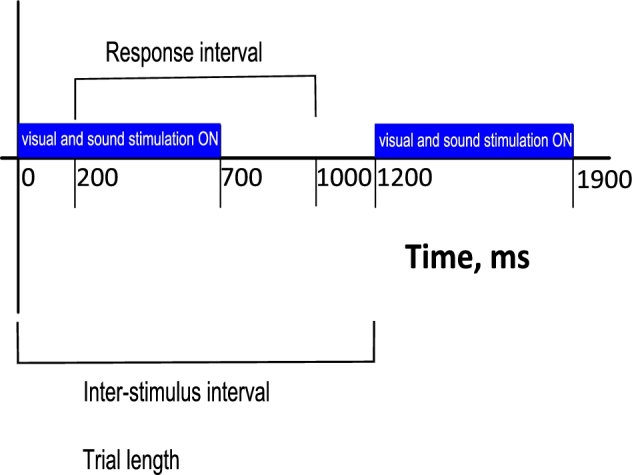
Course of the experiment. Each stimulation marker was associated with 700 ms of sound and visual stimulation. Subsequently, 500 ms without stimulation followed. Therefore, inter-stimulus interval was 1200 ms. The responses of the subjects were considered on time between 200 and 1000 ms after each stimulus.

The inter-stimulus interval (ISI) was 1200 ms, the response interval was between 200 and 1000 ms after each stimulus, and the trial length was set to 1200 ms. Given the number of stimuli and ISI, the total testing time for each run was approximately 6–7 minutes. Fig. 3 depicts the course of the experiment.

#### Data and metadata

The collected data and metadata were stored in the EEG/ERP Portal. The metadata include, for example:

- weather conditions;
- used hardware;
- start time and end time of the experiment;
- temperature in the laboratory;
- used stimulation protocol (scenario title, description, length, source file);
- information about the participant (gender, age, laterality, diseases, etc.).

In addition, experiment-specific metadata about motoric percentiles [10] and hearing thresholds were stored in separate text files along with the datasets.

Finally, for each experiment, important information about behavioral responses of the participants, including reaction times to each stimulus and average reaction times, is stored in the LOG_multimod folders. In the same folder, there is also a file describing the format of these metadata.

### Data validation

**Figure 4:**
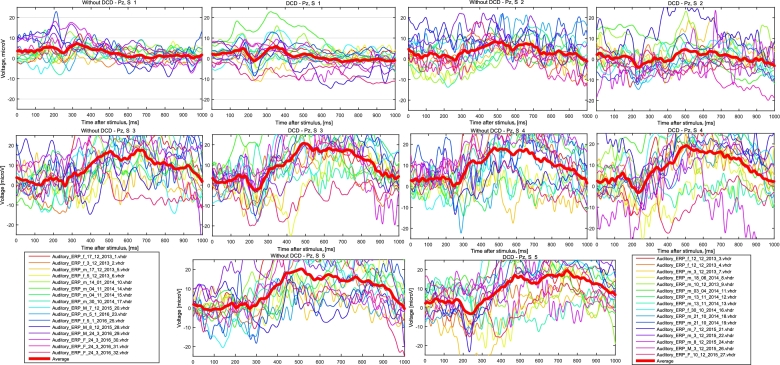
Averages for each participant and each stimulus marker are shown. Figures are divided into two groups based on the condition of the participants (i.e., with DCD/without DCD). Grand averages for each marker are depicted by a **bold red line**. The Pz channel was averaged. Markers used are explained in detail in the attached metadata. S1, standard stimulus (a goat bleats); S2, target stimulus (a dog barks); S3, target stimulus (a cat meows); S4, target stimulus (a cat barks); S5, target stimulus (a dog meows).

First, epochs were averaged for both groups (with and without DCD). The results for the Pz channel are depicted in Fig. 4.

**Figure 5:**
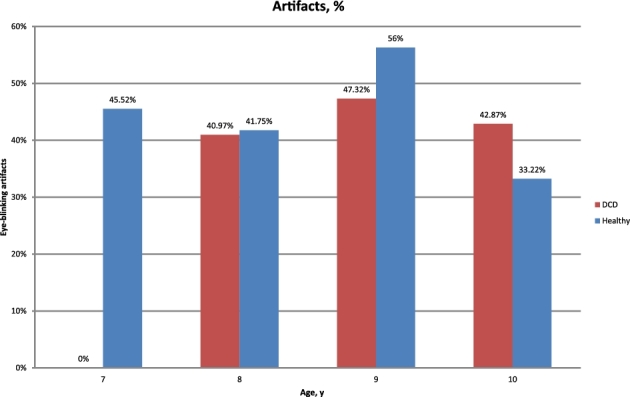
Percentage of eye-blinking artifacts for each age group also divided by the condition of the participants (i.e., with DCD/without DCD).

To evaluate the quality of the data for different subjects, the percentage of eye-blinking artifacts was estimated using visual inspection. The results are depicted in Fig. 5. Although eye blinks cause significant disruptions in the EEG signal, they can be partially corrected using independent component analysis. Therefore, to be able to analyze EEG without excessive data loss even for subjects who blink a lot, independent component analysis, e.g., from EEGLAB or Brain Vision, should be performed.

## Availability of supporting data

Snapshots of the data described here are available under a CC0 waiver from the GigaScience GigaDB repository [11]. The latest experimental data and metadata can also be downloaded from the EEG/ERP Portal [7] according to the following procedure. This has been tested in Internet Explorer 10 and 11, Mozilla Firefox 29.0.1, and Google Chrome. Any user has to be registered first. When the registration form is completed, a confirmation e-mail is sent to the user. Then the user is requested to click on the confirmation link contained in the confirmation e-mail. After a successful login, a personalized user's homepage, including an overview of user's experiments, scenarios, research group memberships, etc., is displayed. In order to see publicly offered experiments and find the package named ‘Developmental coordination disorder in children – experimental work and data annotation,’ the user selects the Experiments section from the main menu appearing at the top of the homepage. When the Experiment section is loaded, the user selects the package ‘Developmental coordination disorder in children – experimental work and data annotation,’ chooses the license under which he/she wants to use the data (Creative Commons BY-NC is the default), and clicks on the ‘Add to cart’ link (free of charge).

When the package is added to the cart, the user is requested to click on the ‘My cart’ link at the top of the page. The experiments in the selected package are available under the selected license. When the user finishes the order (by clicking on the ‘Create order’ button), the download page finally appears (by clicking on the ‘Download’ link). Then the user confirms his/her selection of the experiments within the package and clicks on the ‘Create package’ button to create a zip package. Since the data are quite large, the progress bar indicates the portion of the package that has been created. When the package is created, it can be downloaded by clicking on the ‘Download’ link.

The ordered (purchased) package can be re-downloaded at any time in the Experiment section by clicking on the ‘Download’ link that appears instead of the ‘Add to cart’ link within the package.

## Supplementary Material

GIGA-D-16-00094_Original_Submission.pdf

GIGA-D-16-00094_Revision_1.pdf

GIGA-D-16-00094_Revision_2.pdf

GIGA-D-16-00094_Revision_3.pdf

Response_to_reviewer_comments_Original_Submission.pdf

Response_to_reviewer_comments_Revision_1.pdf

Response_to_reviewer_comments_Revision_2.pdf

Reviewer_1_Report_(Original_Submission).pdf

Reviewer_2_Report_(Original_Submission).pdf

Reviewer_2_Report_(Revision_1).pdf

## Abbreviations

DCD, developmental coordination disorder; EEG, electroencephalography; ERP, event-related potentials; INCF, International Neuroinformatics Coordinating Facility; URL, Uniform Resource Locator.
