# Supplementary material for: Developmental coordination disorder in children – experimental work and data annotation
Source: Gigascience. 2017 Feb 24;6(4):1–6. doi: 10.1093/gigascience/gix002 (PMC5530316; doi:10.1093/gigascience/gix002)
Supplement: GIGA-D-16-00094_Original_Submission.pdf [file gix002_GIGA-D-16-00094_Original_Submission.pdf]

Manuscript (pdf)- PDF version Click here to download Manuscript 8895181815589957_manuscript.pdf

RESEARCH

Developmental coordination disorder in children - experimental work and data annotation

Lukas Vareka[1*], Petr Bruha[1]
, Roman Moucek[1], Pavel Mautner[1], Ladislav Cepicka[1] and Irena Holeckova[2]

*Correspondence:
lvareka@kiv.zcu.cz
[1]University of West Bohemia, Univerzitni 8, 306 14, Plzen, Czech Republic
Full list of author information is available at the end of the article

Abstract

Background: Developmental coordination disorder (DCD) is described as a motor skill disorder characterized by a marked impairment in the development of motor coordination abilities that significantly interferes with performance of daily activities and/or academic achievement [1]. Since some electrophysiological studies suggest differences between children with/without motor development problems [2], we prepared an experimental protocol and performed electrophysiological experiments with the aim to make a step towards a possible diagnosis of this disorder using the event-related potentials (ERP) technique. The second aim is to properly annotate the obtained raw data with relevant metadata and promote their long term sustainability.

Findings: The presented datasets provide sufficient metadata to allow other researchers to perform analysis. For each experiment, the percentage of trials damaged by blinking artifacts was calculated. This should help researchers to estimate the usability of each dataset for analysis.

Conclusions: The data were collected and annotated respecting the current outcomes of INCF Program on Standards for Data Sharing, Task Force on Electrophysiology and the group developing the Ontology for Experimental Neurophysiology (OEN) [3]. The data with metadata are stored in the EEG/ERP Portal [4]. The experimental data and metadata will be also provided in the HDF5 format [5].

Keywords: developmental coordination disorder; event-related potentials; visual and audio stimulation; electroencephalography; reaction time

1 Data description

1.1 Purpose of the study

The degree of motor development is usually assessed through clinical tests such as Movement Assessment Battery for Children (MABC-2) [6]. Our objective was to design and perform event-related potential experiments that can potentially replace traditional behavioral techniques for DCD diagnosis.

1.2 Experimental design

1.2.1 Recording Hardware

The standard 10-20 system EEG cap made by Electro-Cap International was used for the experiment. The EEG cap contained 19 electrodes. The BrainAmp DC amplifier was used with the sampling frequency set to 1 kHz. There were two buttons placed at the armrests of the chair for measuring reactions of participants.

1.2.2 Recording Software

The BrainVision Recorder 1.2 [7] was used for recording and storing the EEG/ERP data in the BrainVision format. The impedance threshold was set to 10 $k\Omega$; the real impedances for each experiment were stored in vhdr files. The Presentation software [8], version 16.3 made by the Neurobehavioral Systems was used for stimulation.

1.2.3 Environment

All experiments were performed in a sound and electrically shielded booth placed in an electrophysiology lab. EEG/ERP activity was recorded using the standard 10-20 international system with the reference electrode placed at the root of the nose.

1.2.4 Stimulation protocol

The experimental protocol was based on multimodal stimulation, i.e. a combination of auditory and visual stimulation. The visual stimuli were represented by pictures of animals. The corresponding auditory stimuli were represented by sounds of the animals that occurred in synchronization with the visual stimuli. One of the pictures (a goat), occurring with the probability of 70%, was always associated with the correct sound, and was the standard (non-target) stimulus. In rare stimuli, the sounds might be incorrectly associated with the animals. The rare stimuli included: barking dog (15%), meowing cat (5%), meowing dog (5%), and barking cat (5%). 600 stimuli were used in total during the experimental session. Each experimental session was divided into two experimental runs, each containing 300 stimuli. During the experimental session, participants were asked to reply to each target stimulus (dog or cat sound) by pressing one button for sounds of barking dog or meowing cat, and the other button for sounds of barking cat or meowing dog.

1.2.5 Participants

The tested subjects were 19 children of younger school age (13 males, 6 females, aged 7-10 years) from a primary school for children with impaired hearing in Pilsen. They were preliminary divided into three groups based on the level of their developmental coordination disorder identified by a motor test. In summary, using the motor test, seven children were diagnosed with DCD, three children had suspected DCD, and seven were healthy. Two children did not participate in the motor test. All children were right-handed, four children had corrected myopia. Most children suffered from hearing impairment. The level of hearing impairment was assessed using a hearing threshold test. The informed consent was signed by their legal guardians.

1.2.6 Procedure

The following experimental procedure was applied:

- Each participant was acquainted with the course of the experiment and answered questions concerning his/her health.
- Each participant was given the headphones. The participant was taken to a soundproof and electrically shielded cabin. The hearing threshold for each ear was evaluated. The volume of auditory stimulation was calculated as follows:

for each ear, the volume was set to be 50 dB higher than the hearing threshold. However, the volume never exceeded 75 dB.

- Each participant was given the standard EEG cap and headphones. 19 electrodes were used as depicted in Fig. 1. The participant was taken to a soundproof and electrically shielded cabin; the reference electrode was placed at the root of his/her nose.
- The participant was told to watch the pictures on the screen, to listen to the sounds, and to respond to stimuli as described in Section 1.2.4.
- The cabin was closed and both the data recording and stimulation started. Fig. 2 shows a participant during the experiment.
- After the experiment had finished, the recorded data and collected metadata were uploaded to the EEG/ERP Portal.

[scale=0.2]figures/TheinternationalStandard10-2019electrode.pdf

Figure 1 The locations of the electrodes.

[scale=0.8]figures/photo_exp.pdf

Figure 2 A participant during the experiment.

1.2.7 Data and metadata

The collected data and metadata were stored in the EEG/ERP Portal. The metadata include, for example:

1 weather conditions
2 used hardware
3 start time and end time of the experiment
4 temperature in the laboratory
5 used stimulation protocol (scenario title, description, length, source file)
6 information about the participant (name, e-mail, gender, date of birth, laterality, diseases)

In addition, experiment-specific metadata about motoric percentiles [9] and hearing thresholds were stored in separate text files along with the datasets. The percentiles at or below 5 indicated DCD. The level between 6 and 15 indicated suspected DCD. The participants scoring more than 15 were considered in the range of typical development [9].

Finally, for each experiment, important information about behavioural responses of the participants, including reaction times to each stimulus and average reaction times, are stored in the LOG_multimod folders. In the same folder, there also is a file describing the format of these metadata.

1.3 Data Validation

To evaluate usability of the data for further analysis, percentage of eye-blinking artifacts for different experiments was calculated using visual inspection. The results are depicted in Fig. 3.

[scale=0.8]figures/artifacts.pdf

Figure 3 Percentage of eye-blinking artifacts for each experiment.

2 Availability and requirements

To download the data described in this article, the following project is available:

- Project name: EEG/ERP Portal [4]
  Project home page: http://eegdatabase.kiv.zcu.cz
  Operating system(s): Platform independent
  Programming language: Java
  Other requirements: tested in Internet Explorer 10, 11, Mozilla Firefox 29.0.1, Google Chrome
  License: GNU GPL

3 Availability of supporting data

The data sets supporting the results of this article are available in the EEG/ERP Portal [4] under the following URL: http://eegdatabase.kiv.zcu.cz/. The experiments described in this paper are associated with the following Experiment IDs $< ID\_EXP >$: 276-278, 280-287, and 289-296.

To download the experimental data and metadata using the EEG/ERP Portal, the user must take the following steps:

- The registration form must be filled out.
- The user is logged in using his/her e-mail address and password.
- The section *Experiments* in the header of the selected page is chosen.
- The "Developmental coordination disorder in children - experimental work and data annotation" package contains the datasets related to this article.
- The data and related metadata can be selected and confirmed after clicking on the *Download* button. By selecting "Choose all", the user can download all the data and metadata related to the specific experiment.

Abbreviations
DCD: Developmental coordination disorder; EEG: electroencephalography; ERP: event-related potentials; INCF: International Neuroinformatics Coordinating Facility; URL: Uniform Resource Locator.

Competing interests
The authors declare that they have no competing interests.

Author's contributions
IH, LC and PM designed the experiments. PB, PM and LC performed the experiments. LV designed data the validation method and analyzed the data. PB prepared datasets for storing. LV, RM and PB wrote the paper. All authors read and approved the final manuscript.

Acknowledgements
This work was supported by the Grant Agency of the Czech Republic under the grant P407/12/1525, by the European Regional Development Fund (ERDF), Project "NTIS - New Technologies for Information Society", European Centre of Excellence, CZ.1.05/1.1.00/02.0090 and by the UWB grant SGS-2013-039 Methods and Applications of Bio- and Medical Informatics.

Author details
[1]University of West Bohemia, Univerzitni 8, 306 14, Plzen, Czech Republic. [2]University Hospital Plzen, alej Svobody 80, 304 60, Plzen, Czech Republic.

References

1. Association, A.P.: Developmental coordination disorder. In: Diagnostic and Statistical Manual of Mental Disorders
2. Monastra, V.J., Lubar, J.F., Linden, M.: The development of a quantitative electroencephalographic scanning process for attention deficit-hyperactivity disorder: reliability and validity studies. Neuropsychology **15**(1), 136–144 (2001)
3. Bruha, P., Papez, V., Bandrowski, A., Grewe, J., Moucek, R., Tripathy, S., Wachtler, T., Le Franc, Y.: The ontology for experimental neurophysiology: a first step toward semantic annotations of neurophysiology data and metadata. Frontiers in Neuroinformatics (26). doi:10.3389/conf.fninf.2013.09.00026
4. Moucek, R., Jezek, P.: EEG/ERP Portal. http://eegdatabase.kiv.zcu.cz/
5. The HDF Group: Hierarchical Data Format, Version 5. http://www.hdfgroup.org/HDF5/
6. Henderson, S.E., Sugden, D.D.A.., Barnett, A.L., Corporation, P.: Movement Assessment Battery for Children-2. London : Harcourt Assessment. Title from Examiner's manual cover (2007)
7. BrainProducts: Brain Vision Recorder. www.brainproducts.com/productdetails.php?id=21
8. NeurobehavioralSystems: Presentation. http://www.neurobs.com/
9. Gueze, R.H., Jongmans, M.J., Schoemaker, M.M., Smits-Engelsman, B.C.: Clinical and research diagnostic criteria for developmental coordination disorder: a review and discussion. Hum Mov Sci **20**(1-2), 7–47 (2001)

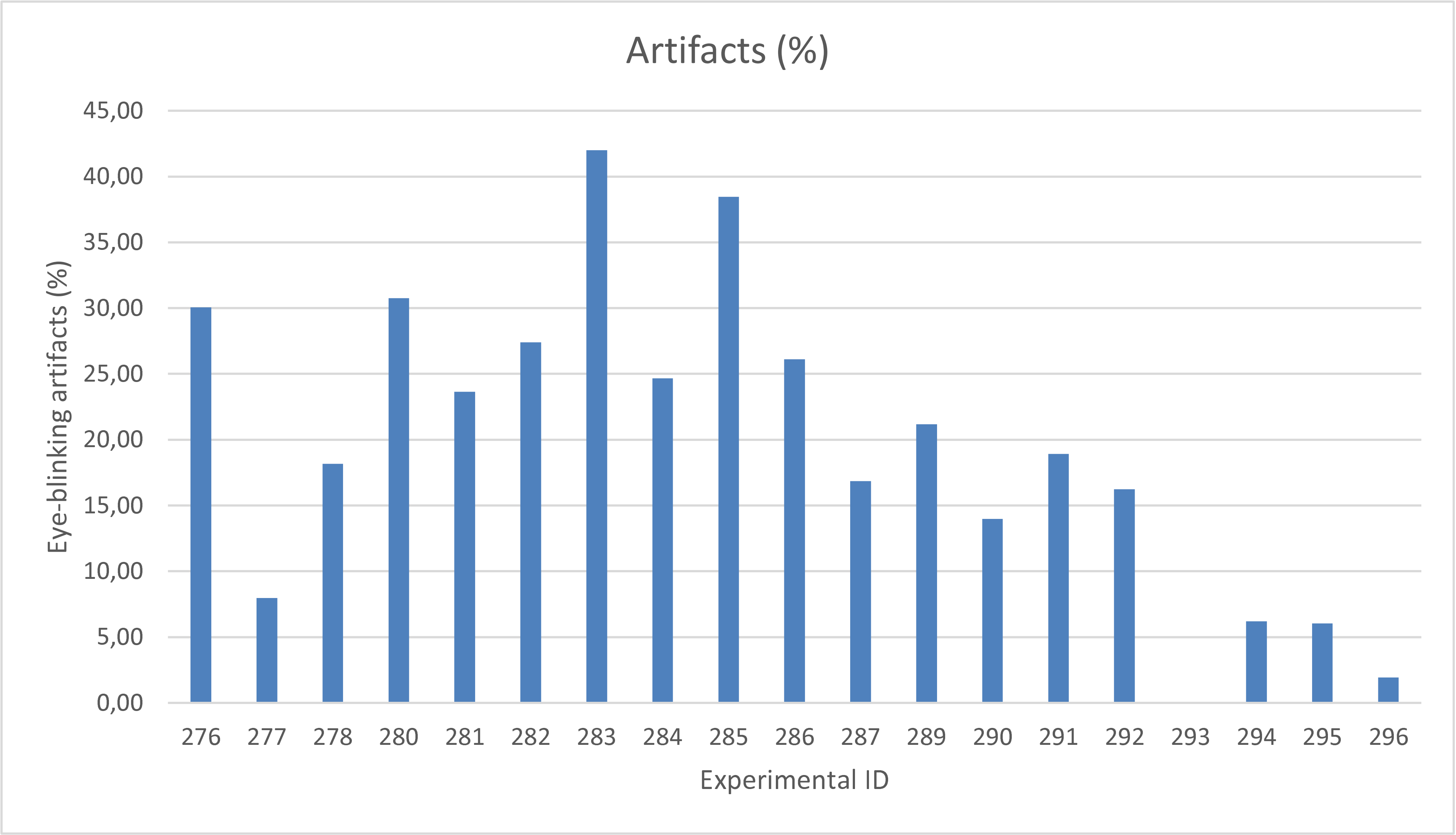
Artifacts (%)
Eye-blinking artifacts (%)
45,00
40,00
35,00
30,00
25,00
20,00
15,00
10,00
5,00
0,00
276
277
278
280
281
282
283
284
285
286
287
289
290
291
292
293
294
295
296
Experimental ID

Figure 1

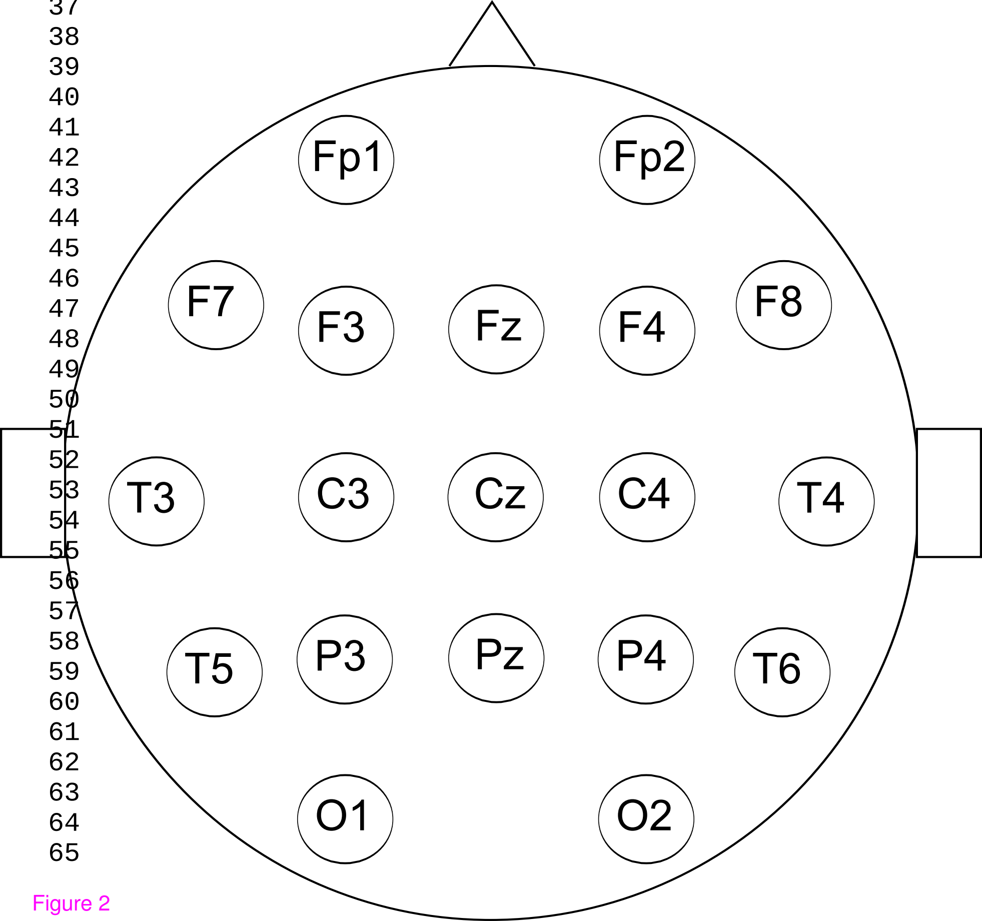

Figure 2

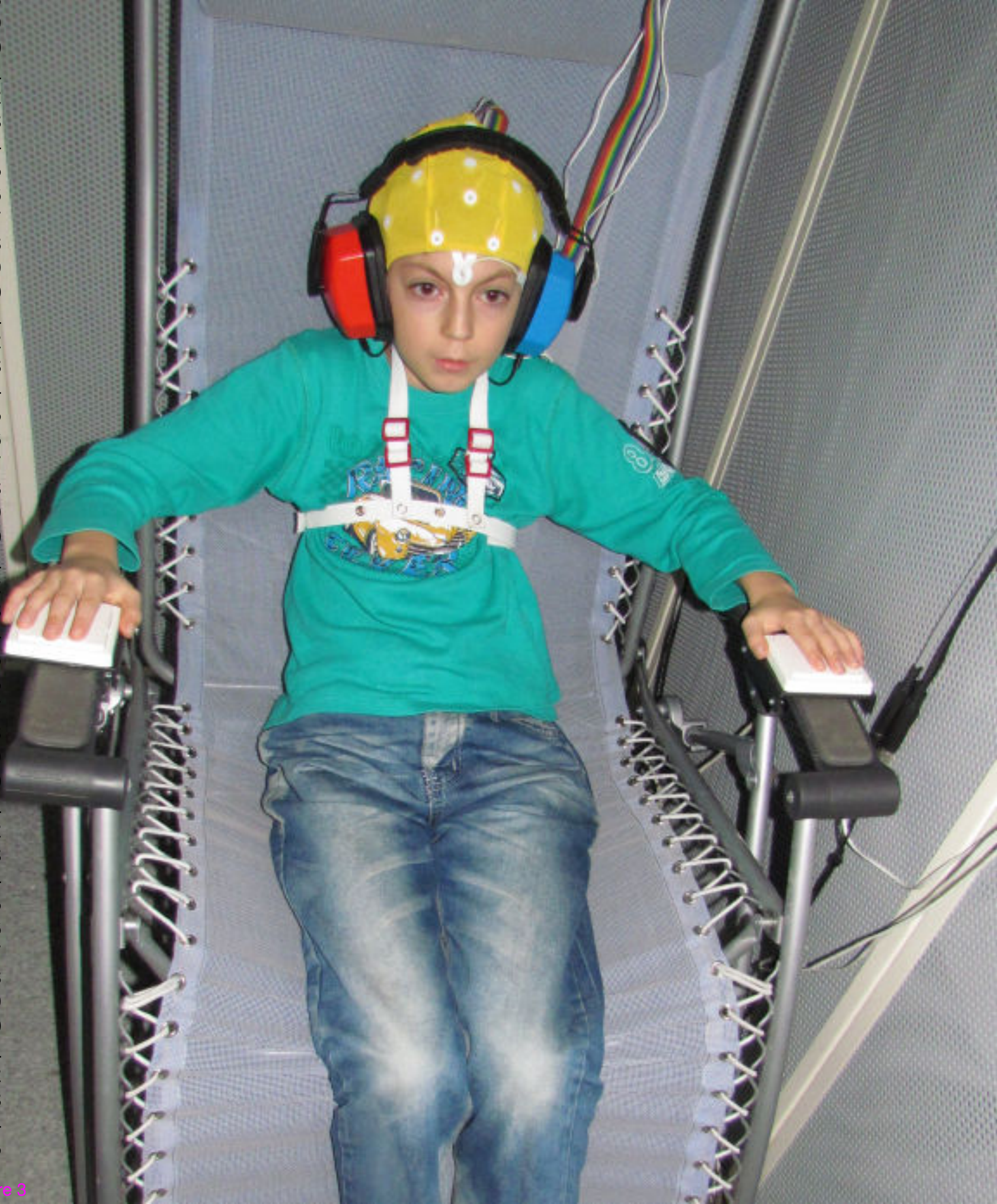

Figure 3

Additional files provided with this submission:

Additional file 1: children_DCD.tex, 22K
http://www.gigasciencejournal.com/imedia/8309770581558990/supp1.tex
Additional file 2: children_DCD.bbl, 8K
http://www.gigasciencejournal.com/imedia/1400206207155899/supp2.bbl

Click here to download Figure fig1.pdf

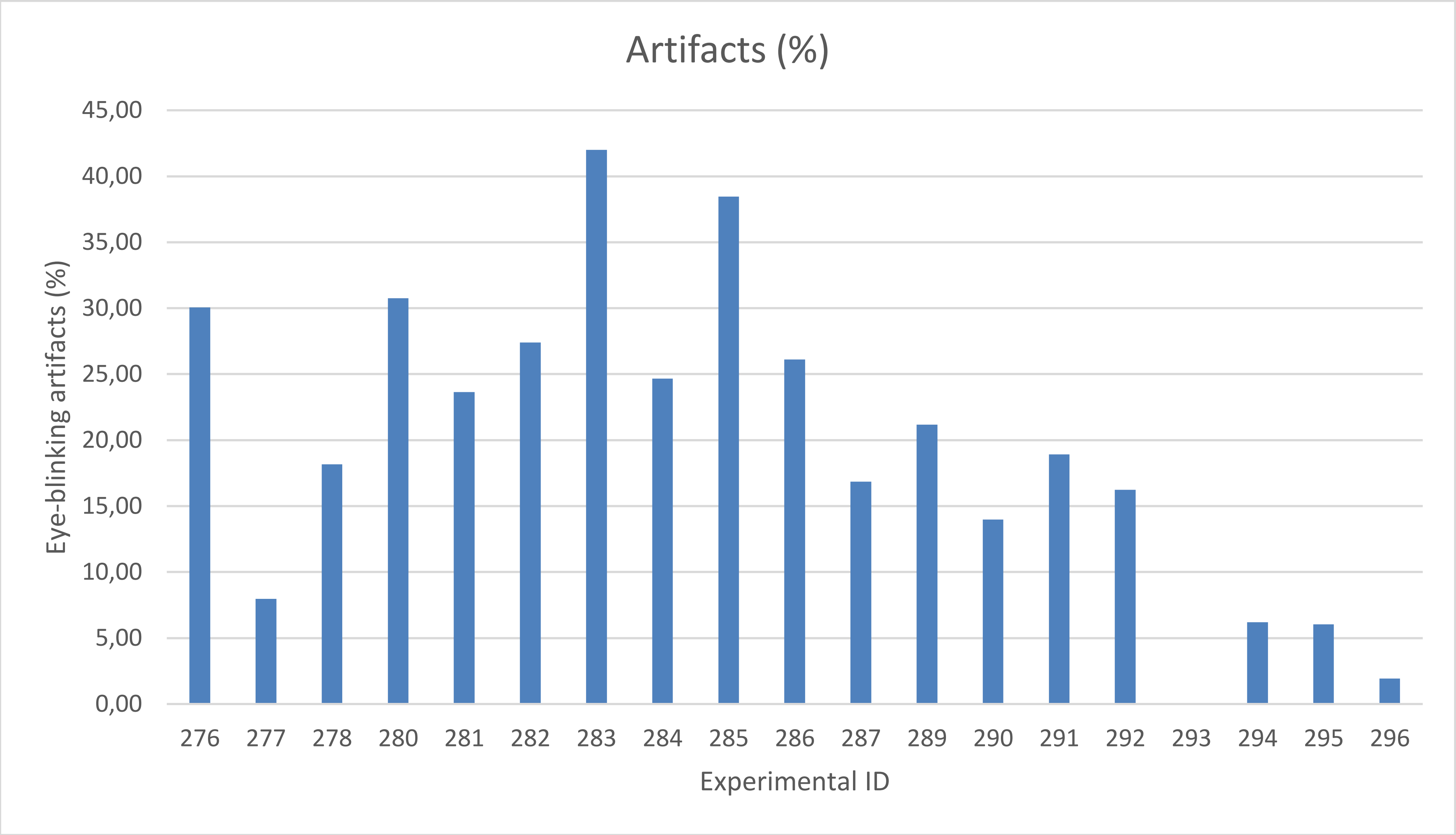

TheinternationalStandard10-2019electrode

Click here to download Figure fig2.pdf

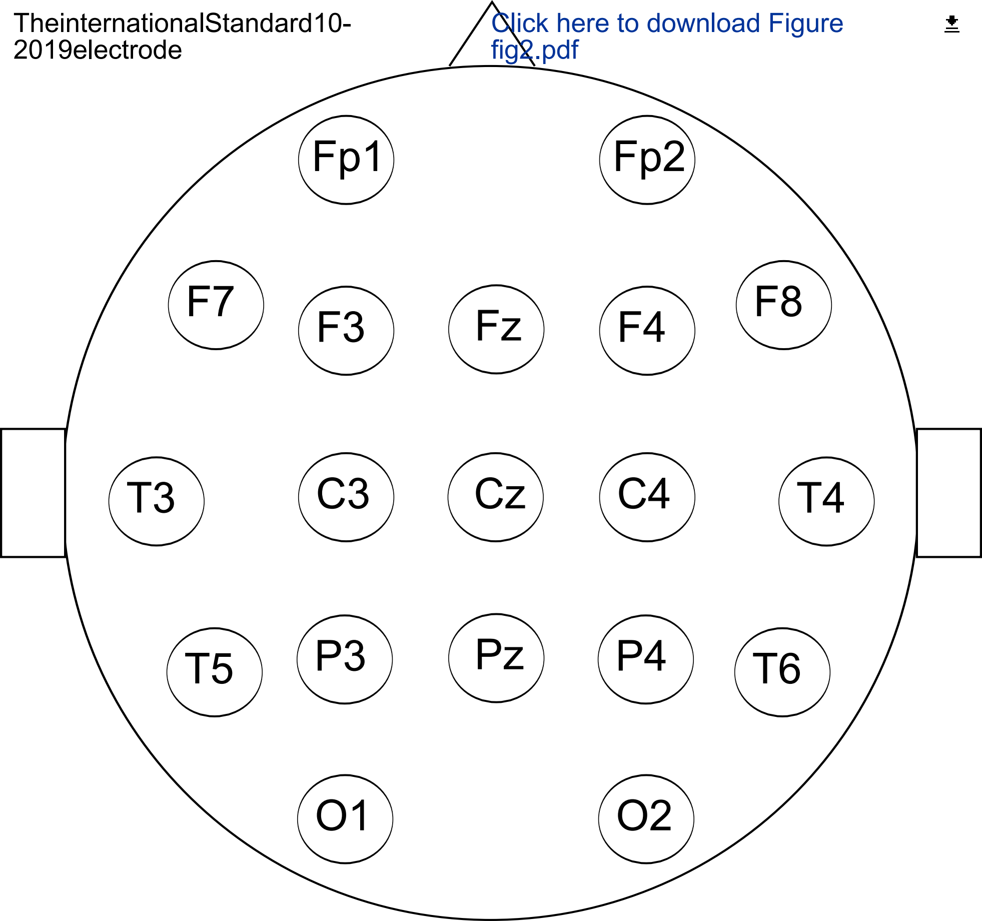

photo_exp

Click here to download Figure fig3.pdf

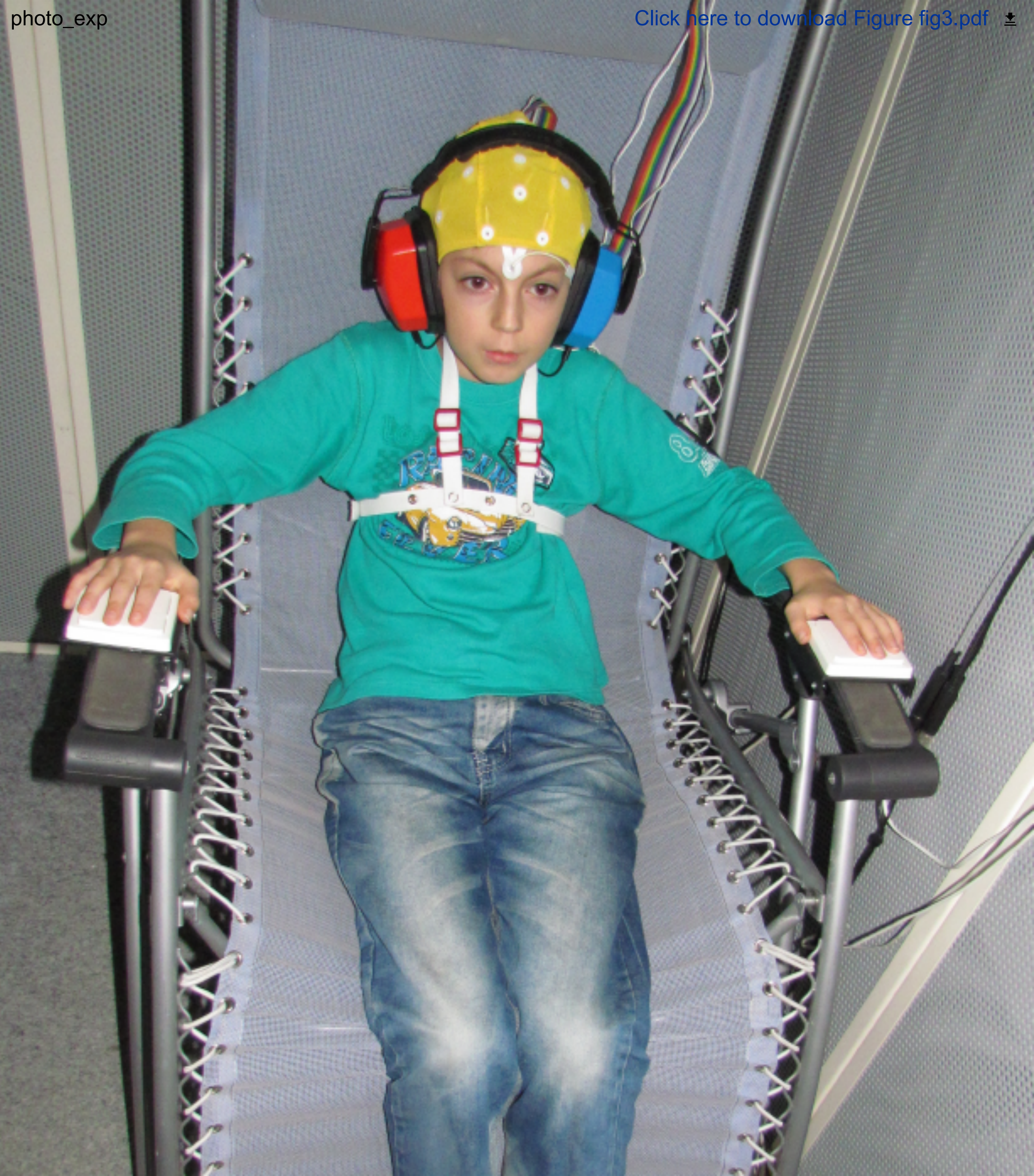

Manuscript without images

Click here to access/download
Supplementary Material
ms.pdf

Source files- Tex file

Click here to access/download
Supplementary Material
ms.tex

Source files- bbl file

Click here to access/download
Supplementary Material
dep1.bbl

Source files-rtf file

Click here to access/download
Supplementary Material
man2183255015525607_ver1.rtf

Personal Cover (comment) Click here to download Personal Cover 1659805280155314_comment (1).pdf

Author's covering letter for initial submission

Title: Developmental coordination disorder in children - experimental work and data annotation

Authors:

Version: 1 **Date:** 30 December 2014

Comments: see over

Lukáš Vařeka, Petr Brůha, Roman Mouček, Pavel Mautner, Ladislav Čepička, Irena Holečková
Corresponding author:
Lukáš Vařeka, lvareka@kiv.zcu.cz
Department of Computer Science and Engineering
University of West Bohemia
Univerzitni 8
306 14 Plzen
Czech Republic
+420 377 632 476

Cover letter

Dear editor,

We are submitting a manuscript entitled “Developmental coordination disorder in children - experimental work and data annotation” to GigaScience journal exclusively. We further note that a part of this work was presented as a poster at the Neuroinformatics 2014 Congress in Leiden, the Netherlands.

Since 2005 we operate a neuroinformatics laboratory where mostly electrophysiological experiments are conducted. The presented experimental work has been done and annotated data have been collected within the research project funded by the Grant Agency of the Czech Republic „Selected parameters of brain functions in relation to developmental coordination disorder in children.“

Our research objective was to design and perform event-related potential experiments that can potentially replace traditional behavioral techniques for DCD diagnosis. The aim of the submitted article is to provide original experimental data and associated metadata to other researchers for further analysis. Also we would like to get feedback from them that is related to the completeness of the set of provided metadata (and thus reproducibility of research) and potentially to get feedback related to usability of the EEG/ERP Portal where the datasets are provided.

We declare that we have no competing interests.

Sincerely

Lukáš Vařeka, Petr Brůha, Roman Mouček, Pavel Mautner, Ladislav Čepička, and Irena Holečková
