# Supplementary material for: Developmental coordination disorder in children – experimental work and data annotation
Source: Gigascience. 2017 Feb 24;6(4):1–6. doi: 10.1093/gigascience/gix002 (PMC5530316; doi:10.1093/gigascience/gix002)
Supplement: GIGA-D-16-00094_Revision_1.pdf [file gix002_GIGA-D-16-00094_Revision_1.pdf]

Manuscript

Click here to download Manuscript 2183255015525607-revised MS.pdf

Click here to view linked References

RESEARCH

Developmental coordination disorder in children - experimental work and data annotation

Lukas Vareka[1*], Petr Bruha[1]
, Roman Moucek[1], Pavel Mautner[1], Ladislav Cepicka[1] and Irena Holeckova[2]

*Correspondence:
lvareka@kiv.zcu.cz
[1]University of West Bohemia, Univerzitni 8, 306 14, Plzen, Czech Republic
Full list of author information is available at the end of the article

Abstract

Background: Developmental coordination disorder (DCD) is described as a motor skill disorder characterized by a marked impairment in the development of motor coordination abilities that significantly interferes with performance of daily activities and/or academic achievement. Since some electrophysiological studies suggest differences between children with/without motor development problems, we prepared an experimental protocol and performed electrophysiological experiments with the aim to make a step towards a possible diagnosis of this disorder using the event-related potentials (ERP) technique. The second aim is to properly annotate the obtained raw data with relevant metadata and promote their long term sustainability.

Findings: The data from 32 school children (16 with possible DCD and 16 in the control group) were collected. Each dataset contains raw EEG data in the BrainVision format and provides sufficient metadata (such as age, gender, results of the motor test, and hearing thresholds) to allow other researchers to perform analysis. For each experiment, the percentage of ERP trials damaged by blinking artifacts was estimated. Furthermore, ERP trials were averaged across different participants and conditions, and the resulting plots are included in the manuscript. This should help researchers to estimate the usability of individual datasets for analysis.

Conclusions: The aim of the whole project is to find out if it is possible to make any conclusions about DCD from EEG data obtained. For the purpose of further analysis, the data were collected and annotated respecting the current outcomes of INCF Program on Standards for Data Sharing, Task Force on Electrophysiology and the group developing the Ontology for Experimental Neurophysiology (OEN). The data with metadata are stored in the EEG/ERP Portal.

Keywords: developmental coordination disorder; event-related potentials; visual and audio stimulation; electroencephalography; reaction time

1 Data description

1.1 Theoretical background and purpose of the study

The degree of motor development is usually assessed through clinical tests such as Movement Assessment Battery for Children (MABC-2) [1]. Since standard motors test are relatively time-consuming and physically demanding, there is an open question whether this disorder can be diagnosed using other techniques, such as electroencephalography (EEG) or event-related potentials (ERP). Different studies have been published that investigate link between EEG and DCD. For example,

in [2], the authors suggest that spectral coherence of certain brain rhythms between different brain regions occurs in children with DCD.

It has been demonstrated that children with DCD have a limited ability to distinguish size, angles, area, and shape compared to children with normal development. Visuospatial processing disorders can be studied using the ERP-based protocol. Furthermore, the high comorbidity [3] between Attention Deficit Hyperactivity Disorder (ADHD) and DCD suggests a possibility of a common developmental anomaly of both disorders. Studies of ERP (in [4] and in [5]) confirmed an attention deficit for both visual and auditory stimuli in children with ADHD. Therefore, given the expected common anomaly in ADHD and DCD, children with DCD should have not only visuospatial attention deficit but also an auditory attention disorder. [3]

Our objective was to design and perform event-related potential experiments that can potentially replace traditional behavioral techniques for DCD diagnosis.

1.2 Participants

The tested subjects were 32 children of younger school age (21 males, 11 females, aged 7-10 years) from a primary school for children with impaired hearing in Pilsen. They were preliminary divided into three groups based on the level of their developmental coordination disorder identified by the MABC-2 motor test [1]. The test evaluates motor performance on three main components: manual dexterity, aiming and catching and balance. The decision was based on the total test score (also referred to as "sum SS") according to a simple Traffic Light system that was proposed in [1]. Children with any score above 67 were in the green zone (no movement difficulty detected). The children that scored between 57 and 67 inclusive were in the yellow zone (at risk of having a movement difficulty). Finally, scores ≤ 56 denoted significant movement difficulty. However, because of a relatively small number of children in the yellow zone, for the purposes of further validation, we decided to merge the yellow zone and the red zone to achieve a group of children with or at risk of DCD. In summary, using the motor test, 16 children were at risk or suffering from DCD (4 out of them were previously in the yellow zone), and 16 were without movement difficulties. All children were right-handed, four children had corrected myopia. Most children suffered from hearing impairment. The level of hearing impairment was assessed using a hearing threshold test. The informed consent was signed by their legal guardians. All participants with some of the important metadata are listed in Table 1.

1.3 Experimental Procedure

The following experimental procedure was applied:

- Each participant was acquainted with the course of the experiment and answered questions concerning his/her health.
- Each participant was given the headphones. The participant was taken to a soundproof and electrically shielded cabin. The hearing threshold for each ear was evaluated. The volume of auditory stimulation was calculated as follows: for each ear, the volume was set to be 50 dB higher than the hearing threshold. However, the volume never exceeded 75 dB.

Table 1 List of all measured participants. Some of the most important metadata are included (VI - Visual impairment, HT - Hearing threshold).

EEGbase ID	Gender	Age	VI	HT (db/1kHz)		MABC-2			Eye-blinks
				left ear	right ear	sum SS	SS	percentile	(%)
276	F	8	no	-5	5	77	9	37	50.40
277	F	7	no	-5	5	72	8	25	27.79
278	F	9	no	0	0	55	5	5	37.05
280	F	10	no	5	5	55	5	5	44.82
281	M	8	no	20	20	74	9	37	37.54
282	F	9	myopia	25	25	73	9	37	43.26
283	M	8	myopia	0	-5	54	5	5	57.48
284	M	8	no	5	5	61	6	9	40.73
285	M	9	no	20	20	65	7	16	57.97
286	M	8	no	15	15	88	12	75	38.94
287	M	10	no	5	20	54	5	5	18.21
289	M	8	no	5	5	43	3	1	43.04
290	M	8	myopia	10	0	54	5	5	29.42
291	M	8	myopia	5	10	85	11	63	37.01
292	M	7	no	20	25	70	8	25	25.40
293	F	7	no	20	20	39	3	1	0.00
294	M	7	no	5	0	73	9	37	30.96
295	M	8	no	20	20	59	6	9	26.71
296	M	8	no	0	-5	47	4	2	14.07
795	M	10	no	5	5	77	9	37	33.22
796	M	10	no	5	10	56	5	5	65.96
797	M	10	no	5	0	42	3	1	42.50
798	M	7	no	15	0	80	10	50	40.72
799	M	8	no	5	0	54	5	5	62.88
800	F	7	no	5	5	68	8	25	65.40
801	M	9	no	0	0	63	7	16	57.59
802	F	8	no	20	25	49	2	4	53.45
803	M	7	no	15	5	71	8	25	67.51
804	M	9	no	5	0	93	14	91	67.69
805	F	7	no	20	20	75	9	37	60.89
806	F	8	no	10	10	85	11	63	39.57
807	F	8	no	5	5	97	15	95	47.03

- Each participant was given a standard 10-20 system EEG cap and headphones. 19 electrodes were used as depicted in Fig. 1. The participant was taken to a soundproof and electrically shielded cabin; the reference electrode was placed at the root of his/her nose.
- The participant was told to watch the pictures on the screen, to listen to the sounds, and to respond to stimuli as described in Section 1.4.4.
- The cabin was closed and both the data recording and stimulation started. Fig. 2 shows a participant during the experiment.
- After the experiment had finished, the recorded data and collected metadata were uploaded to the EEG/ERP Portal. [6]

[scale=0.2]figures/TheinternationalStandard10-2019electrode.pdf

Figure 1 The locations of the electrodes attached in the 10-20 system.

[scale=0.8]figures/photo_exp.pdf

Figure 2 A participant during the experiment.

1.4 EEG data recording

1.4.1 Recording Hardware

The standard 10-20 system EEG cap made by Electro-Cap International was used for the experiment. The EEG cap contained 19 electrodes. The BrainAmp DC amplifier was used with the sampling frequency set to 1 kHz. The raw signal was filtered using an analogue band-pass filter with the cut-off frequencies of 0.1 and 250 Hz. There were two buttons placed at the armrests of the chair for measuring reactions of participants (also depicted in Fig. 2).

1.4.2 Recording Software

The BrainVision Recorder 1.2 [7] was used for recording and storing the EEG/ERP data in the BrainVision format. The impedance threshold was set to 10 $k\Omega$; the real impedances for each experiment were stored in vhdr files. The Presentation software [8], version 16.3 made by the Neurobehavioral Systems was used for stimulation.

1.4.3 Environment

All experiments were performed in a sound and electrically shielded booth placed in an electrophysiology lab. EEG/ERP activity was recorded using the standard 10-20 international system with the reference electrode placed at the root of the nose.

1.4.4 Stimulation protocol

The experimental protocol was based on multimodal stimulation, i.e. a combination of auditory and visual stimulation. The visual stimuli were represented by pictures of animals. The corresponding auditory stimuli were represented by sounds of the animals that occurred in synchronization with the visual stimuli. One of the pictures (a goat), occurring with the probability of 70%, was always associated with the correct sound, and was the standard (non-target) stimulus. In rare stimuli, the sounds might be incorrectly associated with the animals. The rare stimuli included: barking dog (15%), meowing cat (5%), meowing dog (5%), and barking cat (5%). 600 stimuli were used in total during the experimental session. Each experimental session was divided into two experimental runs, each containing 300 stimuli. During the experimental session, participants were asked to reply to each target stimulus (dog or cat sound) by pressing one button for sounds of barking dog or meowing cat, and the other button for sounds of barking cat or meowing dog.

Inter-stimulus interval (ISI) was 1200 ms, response interval between 200 ms and 1000 ms after each stimulus, and trial length was set to 1200 ms. Given the number of stimuli and ISI, the total testing time for each run was approximately 6 - 7 minutes. Fig. 3 depicts the course of the experiment.

[scale=0.8]figures/timeline.pdf

Figure 3 Course of the experiment. Each stimulation marker was associated with 700 ms of sound and visual stimulation. Subsequently, 500 ms without stimulation followed. Therefore, inter-stimulus interval was 1200 ms. The responses of the subjects were considered on time between 200 ms and 1000 ms after each stimulus.

1.4.5 Data and metadata

The collected data and metadata were stored in the EEG/ERP Portal. The metadata include, for example:

1 weather conditions
2 used hardware
3 start time and end time of the experiment
4 temperature in the laboratory
5 used stimulation protocol (scenario title, description, length, source file)
6 information about the participant (gender, age, laterality, diseases, etc.)

In addition, experiment-specific metadata about motoric percentiles [9] and hearing thresholds were stored in separate text files along with the datasets.

Finally, for each experiment, important information about behavioral responses of the participants, including reaction times to each stimulus and average reaction times, are stored in the LOG_multimod folders. In the same folder, there also is a file describing the format of these metadata.

1.5 Data Validation

First, epochs were averaged for both groups (with and without DCD). The results for the Pz channel are depicted in Fig. 4.

[scale=0.7]figures/allcmp19.pdf

Figure 4 Averages for each participant and each stimulus marker are shown. Figures are divided into two groups based on the condition of the participants (i.e. with DCD / without DCD). Grand averages for each marker are depicted in a red bold line. The Pz channel was averaged. Markers used are in detail explained in the attached metadata. S1 – standard stimulus (a goat bleats), S2 - target stimulus (a dog barks), S3 – target stimulus (a cat mews), S4 – target stimulus (a cat barks), S5 – target stimulus (a dog mews).

To evaluate usability of the data for further analysis, percentage of eye-blinking artifacts for different experiments was estimated using visual inspection. The results are depicted in Fig. 5.

[scale=0.8]figures/artifactsAge.pdf

Figure 5 Percentage of eye-blinking artifacts for each age group also divided by the condition of the participants (i.e. with DCD / without DCD).

2 Availability and requirements

To download the data described in this article, the following project is available:

- Project name: EEG/ERP Portal [6]
  Project home page: http://eegdatabase.kiv.zcu.cz
  Operating system(s): Platform independent
  Programming language: Java
  Other requirements: tested in Internet Explorer 10, 11, Mozilla Firefox 29.0.1, Google Chrome
  License: Creative Commons BY-NC-SA 4.0

3 Availability of supporting data

The experimental data and metadata can be downloaded from the EEG/ERP Portal according to the following procedure. Any user has to be registered first. When the registration form is completed, a confirmation e-mail is sent to the user. Then the user is requested to click on the confirmation link contained in the confirmation e-mail. After a successful login a personalized user's homepage including an overview of user's experiments, scenarios, research group memberships, etc. is displayed. In order to see publicly offered experiments and find the package named 'Developmental coordination disorder in children – experimental work and data annotation', the user selects the Experiments section from the main menu appearing at the top of the homepage. When the Experiment section is loaded, the user selects the package 'Developmental coordination disorder in children – experimental work and data annotation', chooses the license under which he/she wants to use the data (Creative Commons BY-NC) and clicks on the 'Add to cart' link (free of charge).

When the package is added into the cart, the user is requested to click on the 'My cart' link at the top of the page. The experiments in the selected package are available under the selected license. When the user finishes the order (by clicking on the 'Create order' button), the download page finally appears (by clicking on the 'Download' link). Then the user confirms his/her selection of the experiments within the package and clicks on the 'Create package' button to create a zip package. Since the data are quite large, the progress bar indicates the portion of the package that has been already created. When the package is created, it can be finally downloaded by clicking on the 'Download' link.

The ordered (purchased) package could be re-downloaded at any time in the Experiment section by clicking on the 'Download' link that appears instead of the 'Add to cart' link within the package.

Abbreviations
DCD: Developmental coordination disorder; EEG: electroencephalography; ERP: event-related potentials; INCF: International Neuroinformatics Coordinating Facility; URL: Uniform Resource Locator.

Competing interests
The authors declare that they have no competing interests.

Author's contributions
IH, LC and PM designed the experiments. PB, PM and LC performed the experiments. LV designed the data validation method and analyzed the data. PB prepared datasets for storing. LV, RM and PB wrote the paper. All authors read and approved the final manuscript.

Acknowledgements
This work was supported by the Grant Agency of the Czech Republic under the grant P407/12/1525.

Author details
[1]University of West Bohemia, Univerzitni 8, 306 14, Plzen, Czech Republic. [2]University Hospital Plzen, Alej Svobody 80, 304 60, Plzen, Czech Republic.

References
1. Henderson, S.E., Sugden, D.D.A.., Barnett, A.L., Corporation, P.: Movement Assessment Battery for Children-2. London : Harcourt Assessment. Title from Examiner's manual cover (2007)
2. de Castelnau, P., Albaret, J.-M., Chaix, Y., Zanone, P.-G.: A study of {EEG} coherence in {DCD} children during motor synchronization task. Human Movement Science **27**(2), 230–241 (2008). doi:10.1016/j.humov.2008.02.006. Developmental Coordination Disorder
3. Holeckova, I., Cepicka, L., Mautner, P., Stepanek, D., Moucek, R.: Auditory erps in children with developmental coordination disorder. Activitas Nervosa Superior, 37–44 (2014)
4. Winsberg, B.G., Javitt, D.C., Silipo, G.S., Doneshka, P.: Mismatch negativity in hyperactive children: effects of methylphenidate. Psychopharmacol Bull **29**(2), 229–233 (1993)

5. Kemner, C., Verbaten, M.N., Koelega, H.S., Buitelaar, J.K., van der Gaag, R.J., Camfferman, G., van Engeland, H.: Event-related brain potentials in children with attention-deficit and hyperactivity disorder: effects of stimulus deviancy and task relevance in the visual and auditory modality. Biol. Psychiatry **40**(6), 522–534 (1996)
6. Moucek, R., Jezek, P.: EEG/ERP Portal. http://eegdatabase.kiv.zcu.cz/
7. BrainProducts: Brain Vision Recorder. www.brainproducts.com/productdetails.php?id=21
8. NeurobehavioralSystems: Presentation. http://www.neurobs.com/
9. Gueze, R.H., Jongmans, M.J., Schoemaker, M.M., Smits-Engelsman, B.C.: Clinical and research diagnostic criteria for developmental coordination disorder: a review and discussion. Hum Mov Sci **20**(1-2), 7–47 (2001)

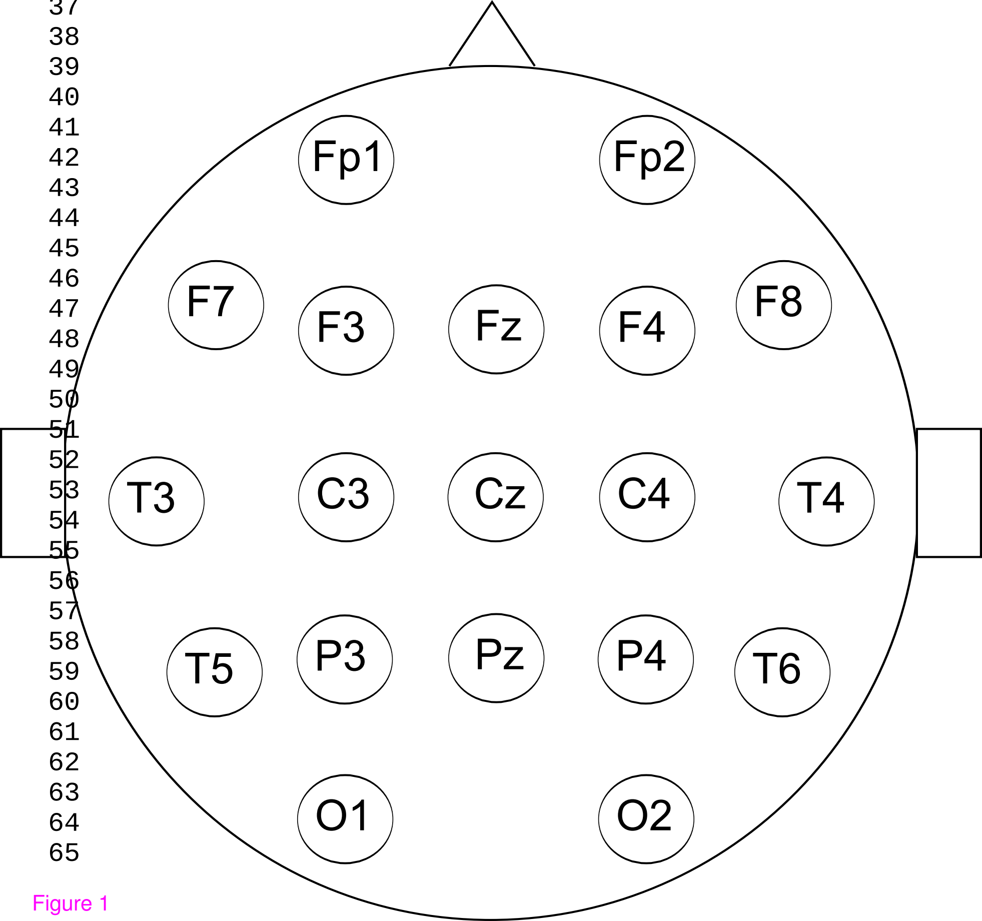

Figure 1

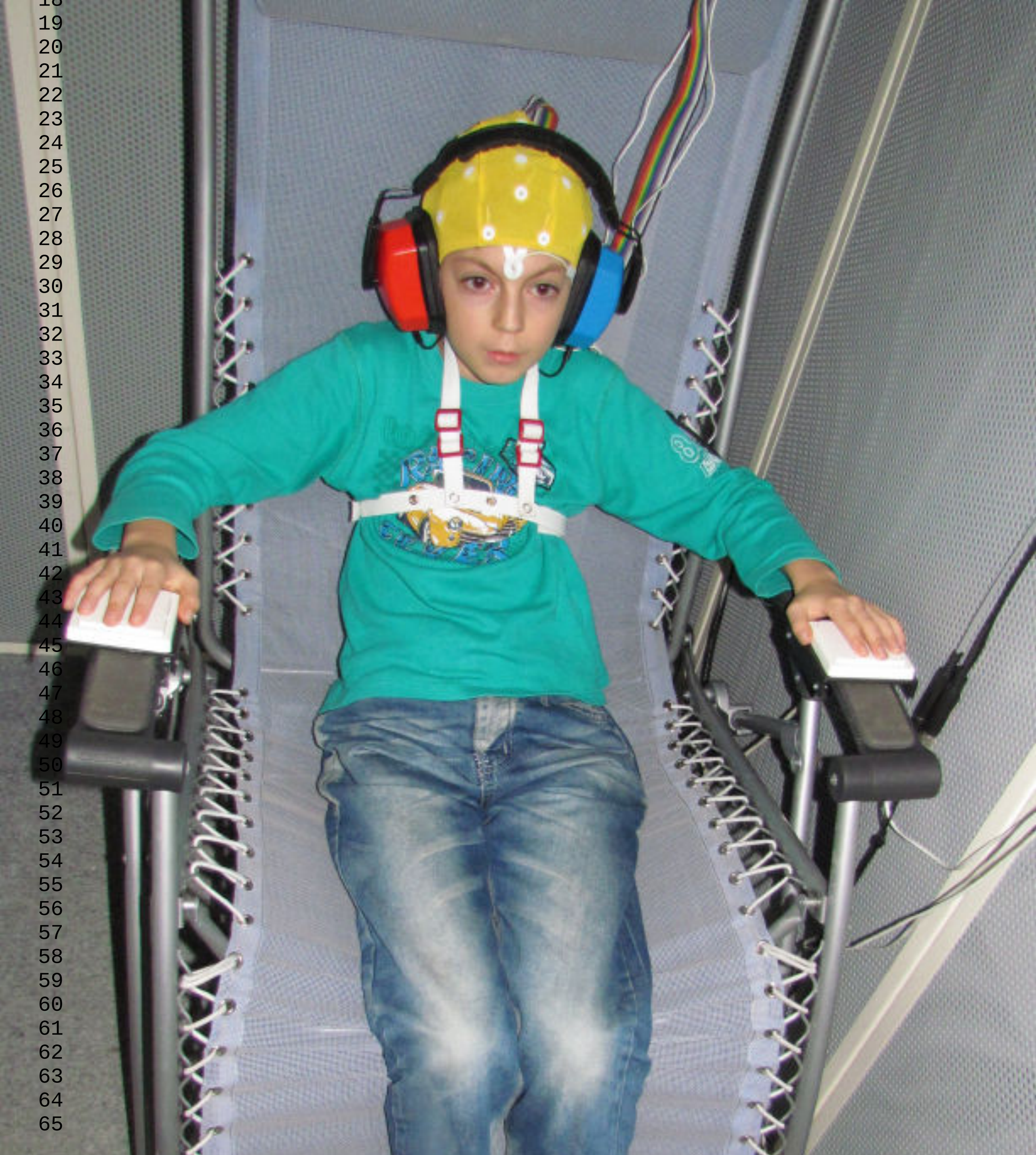

Figure 2

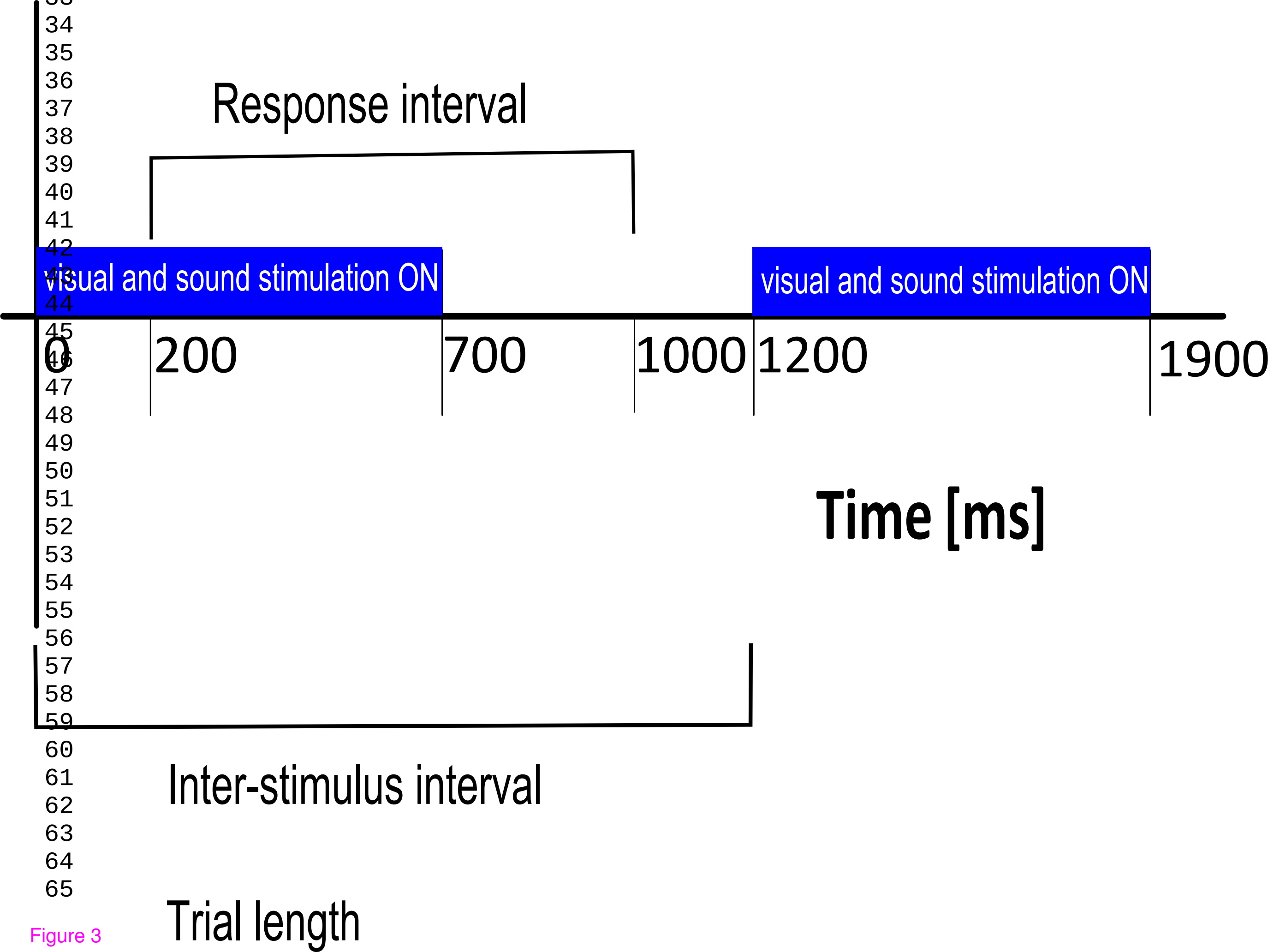
Response interval
visual and sound stimulation ON
visual and sound stimulation ON
0
200
700
1000
1200
1900
Time [ms]
Inter-stimulus interval
Trial length

Figure 3

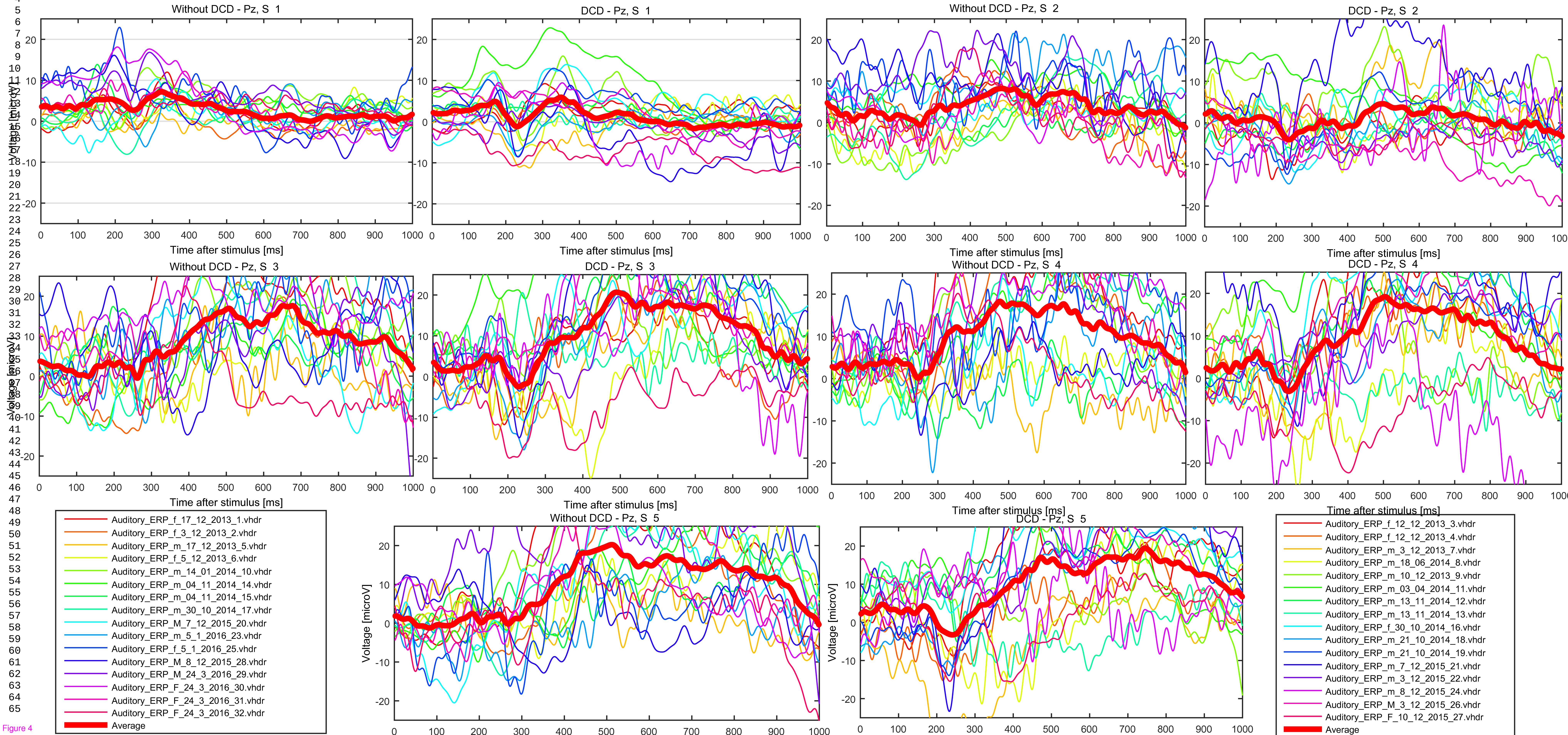

Without DCD - Pz, S 1
DCD - Pz, S 1
Without DCD - Pz, S 2
DCD - Pz, S 2
Without DCD - Pz, S 3
DCD - Pz, S 3
Without DCD - Pz, S 4
DCD - Pz, S 4
Without DCD - Pz, S 5
DCD - Pz, S 5
Voltage [microV]
Time after stimulus [ms]
Auditory_ERP_f_17_12_2013_1.vhdr
Auditory_ERP_f_3_12_2013_2.vhdr
Auditory_ERP_m_17_12_2013_5.vhdr
Auditory_ERP_f_5_12_2013_6.vhdr
Auditory_ERP_m_14_01_2014_10.vhdr
Auditory_ERP_m_04_11_2014_14.vhdr
Auditory_ERP_m_04_11_2014_15.vhdr
Auditory_ERP_m_30_10_2014_17.vhdr
Auditory_ERP_M_7_12_2015_20.vhdr
Auditory_ERP_m_5_1_2016_23.vhdr
Auditory_ERP_f_5_1_2016_25.vhdr
Auditory_ERP_M_8_12_2015_28.vhdr
Auditory_ERP_M_24_3_2016_29.vhdr
Auditory_ERP_F_24_3_2016_30.vhdr
Auditory_ERP_F_24_3_2016_31.vhdr
Auditory_ERP_F_24_3_2016_32.vhdr
Average
Auditory_ERP_f_12_12_2013_3.vhdr
Auditory_ERP_f_12_12_2013_4.vhdr
Auditory_ERP_m_3_12_2013_7.vhdr
Auditory_ERP_m_18_06_2014_8.vhdr
Auditory_ERP_m_10_12_2013_9.vhdr
Auditory_ERP_m_03_04_2014_11.vhdr
Auditory_ERP_m_13_11_2014_12.vhdr
Auditory_ERP_m_13_11_2014_13.vhdr
Auditory_ERP_f_30_10_2014_16.vhdr
Auditory_ERP_m_21_10_2014_18.vhdr
Auditory_ERP_m_21_10_2014_19.vhdr
Auditory_ERP_m_7_12_2015_21.vhdr
Auditory_ERP_m_3_12_2015_22.vhdr
Auditory_ERP_m_8_12_2015_24.vhdr
Auditory_ERP_M_3_12_2015_26.vhdr
Auditory_ERP_F_10_12_2015_27.vhdr
Average

Figure 4

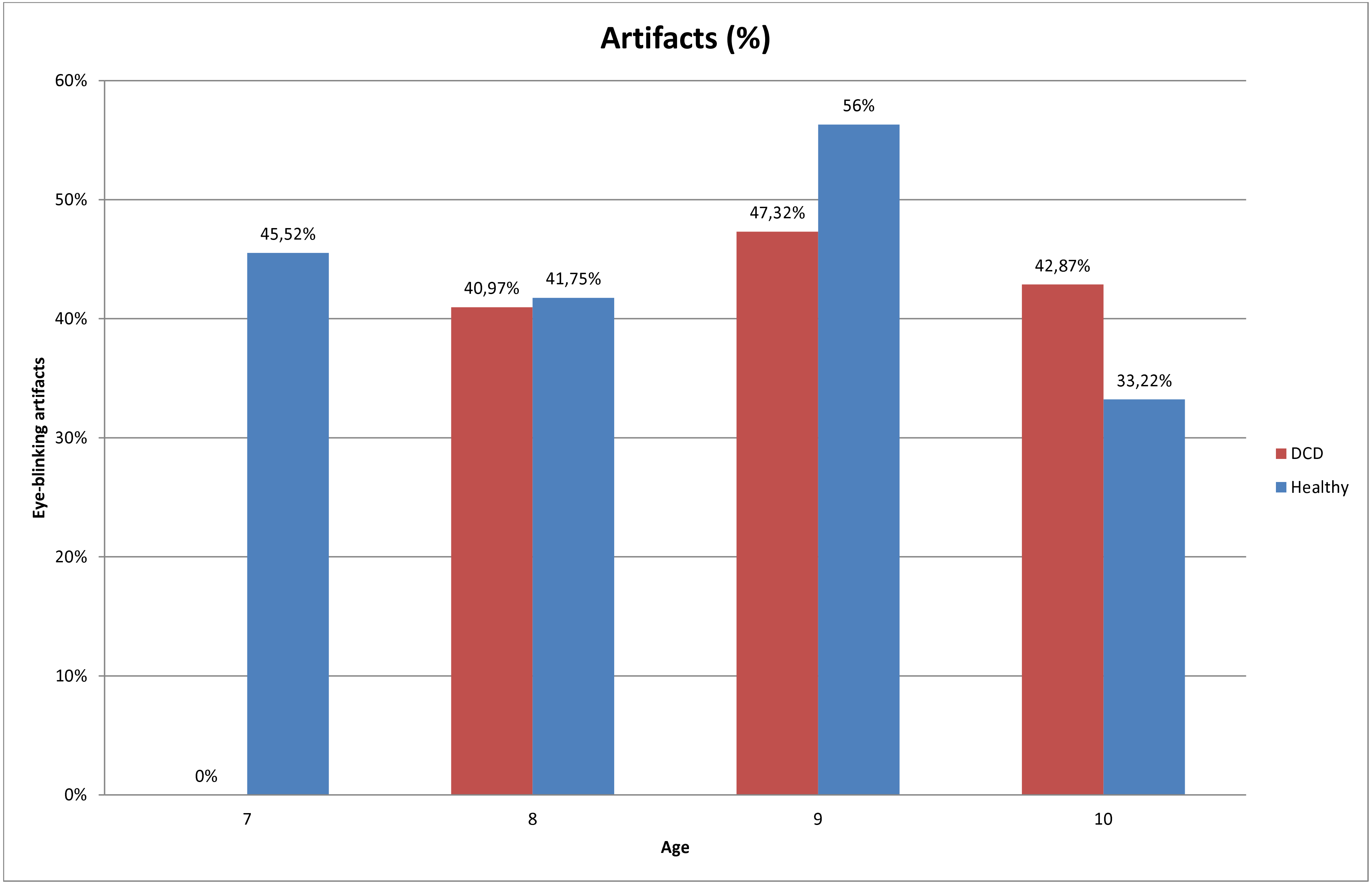
Artifacts (%)
60%
50%
40%
30%
20%
10%
0%
Eye-blinking artifacts
0%
45,52%
40,97%
41,75%
47,32%
56%
42,87%
33,22%
7
8
9
10
Age
DCD
Healthy

Figure 5

Additional files provided with this submission:

Additional file 1: children_DCD.tex, 30K
http://www.gigasciencejournal.com/imedia/4337557532015875/supp1.tex
Additional file 2: children_DCD.bbl, 8K
http://www.gigasciencejournal.com/imedia/1112427527201587/supp2.bbl

Click here to download Personal Cover 2183255015525607-response to reviewers.pdf

Author's response to reviews

Title: Developmental coordination disorder in children - experimental work and data annotation

Authors:

Lukas Vareka (lvareka@kiv.zcu.cz)
Petr Bruha (pbruha@kiv.zcu.cz)
Roman Moucek (moucek@kiv.zcu.cz)
Pavel Mautner (mautner@kiv.zcu.cz)
Ladislav Cepicka (lcepicka@ktv.zcu.cz)
Irena Holeckova (holeckova@fnplzen.cz)

Version: 3 **Date:** 29 June 2016

Author's response to reviews: see over

Dear GigaScience reviewers,

we have tried to address all the points that you mentioned in your review. We have revised our manuscript and below, we are giving a point-by-point response to the concerns.

Thank you for your feedback.

Best regards,
Lukas Vareka,
Petr Bruha,
Roman Moucek

Susanne Passow:

Point 1

A brief section about the theoretical background reviewing some literature in the field is missing. For instance, as a reader it would be interesting what EEG signatures/ features have already been used in previous studies to differentiate between children with and without developmental coordination disorder (DCD), at least 1-2 examples.

New text inserted into the chapter Purpose of the study:
"Since standard motors test are relatively time-consuming and physically demanding, there is an open question whether this disorder can be diagnosed using other techniques, such as electroencephalography (EEG) or event-related potentials (ERP). Different studies have been published that investigate link between EEG and DCD. For example, in~\cite{deCastelnau2008230}, the authors suggest that spectral coherence of certain brain rhythms between different brain regions occurs in children with DCD.

It has been demonstrated that children with DCD have a limited ability to distinguish size, angles, area, and shape compared to children with normal development. Visuospatial processing disorders can be studied using the ERP-based protocol. Furthermore, the high comorbidity~\cite{auditoryERPChildren} between Attention Deficit Hyperactivity Disorder (ADHD) and DCD suggests a possibility of a common developmental anomaly of both disorders. Studies of ERP (in~\cite{pmid8290670} and in~\cite{pmid8879473}) confirmed an attention deficit for both visual and auditory stimuli in children with ADHD. Therefore, given the expected common anomaly in ADHD and DCD, children with DCD should have not only visuospatial attention deficit but also an auditory attention disorder.~\cite{auditoryERPChildren}"

Point 2

It does not come across clearly whether the dataset has been analysed and published before. If yes, please provide a reference, this would be helpful for a potential user. If not, it would still be interesting to know more about the initial aim of the data collection or was the initial aim to provide the dataset for public use.

The dataset has never been published before.

The purpose of the novel dataset subsequently described in this paper is to verify if DCD can be reliably diagnosed using ERP techniques.

Point 3

The section about 'Findings' in the abstract should inform in a more detailed way about the data types provided. The 'Conclusion' should provide more information about potential uses and implications for the research field. Please revise.

The following text was added:

Findings: Each dataset contains raw EEG data in the BrainVision format and provides sufficient metadata (such as age, gender, results of the motor test, and hearing thresholds) to allow other researchers to perform analysis.
Conclusion: The aim of the whole project is to find out if it is possible to make any conclusions about DCD from EEG data obtained.

Point 4

A dataset of N=19, per se, is not particularly large-scale in the field of EEG studies. Further, this dataset is divided into three groups, one group of children with DCD diagnosis (N=7), a group of children with suspected DCD (N=3), and a healthy control group (N=7). In two children motor test data is missing. To detect solid group differences and condition x group interactions, which are desirable when aiming at using the ERP as a diagnostic marker, the present sample size is too small. For instance, aiming for a medium effect size of Cohen's f= 0.25 (#=0.05) and a power of 0.85 for condition x group interaction effects, one would need a total sample size of 48 subjects, thus at least 16 subjects per group. Consequently, I suggest to record additional datasets to increase the utility of the dataset.

We were able to collect additional data to create two groups of 16. The first group was diagnosed with DCD or potentially suffering from milder DCD using the standard motor tests.

The second group was healthy, and can be used as a control group for further statistical processing. Unfortunately, we were not able to collect 3 groups of 16 (DCD, potencial DCD, healthy control group) because subjects in the second group are especially hard to find and the measurements are time-consuming.

Point 5

The definition of the groups is not clear enough in the section about 'Participants'. Please provide the information about the motoric percentiles and the cut-off values for the different groups already here. What was the motor test about? Please provide more details.

The following text has been added: The test evaluates motor performance on three main components: manual dexterity, aiming and catching and balance. The decision was based on the total test score (also referred to as "sum SS") according to a simple Traffic Light system that was proposed in~\cite{MABC-2}. Children with any score above 67 were in the green zone (no movement difficulty detected). The children that scored between 57 and 67 inclusive were in the yellow zone (at risk of having a movement difficulty). Finally, scores <= 56 denoted significant movement difficulty. However, because of a relatively small number of children in the yellow zone, for the purposes of further validation, we decided to merge the yellow zone and the red zone to achieve a group of children with or at risk of DCD.

Point 6

The section about 'Data description' needs to be improved. It is a bit misleading to read something about the recording hardware under a subheading of 'Experimental Design'. Maybe you can apply the following headings and order:

1. **Theoretical background and purpose of the study; 2. Participants; 3. Experimental Procedure; 4. EEG data recording. With respect to information about the EEG data recording please see Point 8.**

The following replacements were applied in the headings:
Purpose of the study -> Theoretical background and purpose of the study
Participants -> Participants (moved forward)
Procedure -> Experimental Procedure (and moved forward)
Experimental Design -> EEG data recording

Point 7

Could you please provide more detailed information about the hearing impairment of the participants? Were all hearing thresholds # 25 dB HL or not? If 75 dB SPL was set as an upper limit and the thresholds were higher 25 dBHL,

some of the children might have gotten higher intensity increments than others. As this is partly an auditory task, this might have influenced the results. Please explain.

This situation occurred for two subjects only. We wanted to prevent hear injuries in case of too high sound intensity.

Was the study approved by an ethical committee? Please provide information.

The study was not approved by an ethical comittee because the university has no ethical comittee processing these matters. However, the experiments were performed within the grant approved by the Grant Agency of the Czech Republic. The parents of all children signed the informed consent.

Point 8

When describing the ERP data recording, you should consider the very useful guidelines provided by Picton et al. 2000 in Psychophysiology facilitating the replicability of ERP results.

Picton, T. W., Bentin, S., Berg, P., Donchin, E., Hillyard, S. A., Johnson, R., . . .

Taylor, M. J. (2000). Guidelines for using human event-related potentials to study cognition: Recording standards and publication criteria. Psychophysiology, 37(2), 127-152.

For instance, I am missing information about the timing of the stimuli (trial length, response interval; inter-stimulus interval) and about breaks between the blocks. More detailed information about the kind of electrodes and the preparation of the EEG should be provided. Further information about the filtering characteristics of the recording system (i.e. use of an online bandpass filter) should be provided. How were the eye blinks determined.

The information required was added into the text of the article.

Point 9

I am not an expert on data security, but is it allowed to provide non-anonymized data as name and e-mail addresses in such a context?

As far as we know, the typical user cannot see any personal data in the EEG/ERP portal, at least not in the current version. The information in the article saying that the non-anonymized data are available, was incorrect.

Point 11

Is the percentage of eye blinks based on the number of total trials, thus 600? More informative would be if the percentage of eye blinks varied significantly across conditions and across groups. Could you provide this information in addition?

The plot reflecting these differences was added.

Minor Essential Revisions

- Abstract: Please do not cite references in the abstract
Corrected.

- Figure 1: Please add in the figure caption that the electrodes have been attached in the 10-20 system.
Corrected.

- Figure 2 is not carrying any important information. I don't think it is necessary.
The figure shows typical conditions during the experiments, especially the placement of response buttons. We would like to have this figure in the article.

- Figure 3: Figure caption should notify that the percentage of eye-blink artifacts is presented for each participant and not for each experiment. Also see my point 11.
The plot was added.

Melissa Pangelinan:

1.

I am not sure if traditional results are necessary in a data note submission but future users would greatly benefit from at least one data visualization to determine if these data are of sufficient quality. A figure with the individual ERPs for each group (not the group grand-average ERPs) for each condition would be particularly useful. This would provide users with an idea of the variability of these data, particularly for the children with DCD.

Both individual averages and grand averages for each condition (possible DCD, control group without DCD) and each stimulus were depicted in Figure 1. However, this figure may be too large to fit into the page.

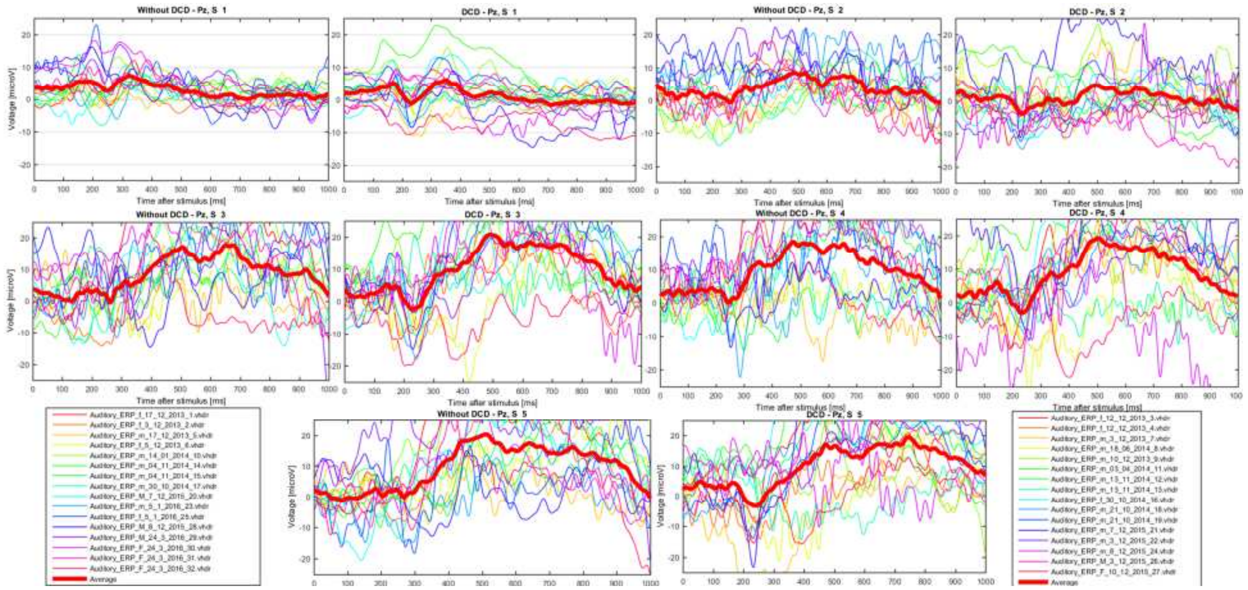

Figure 1. The Pz channel: individual averages (thin lines) and grand averages (bold red) for each stimulus S 1 - S 5 as described in a README file that is attached to data and metadata.

S1 – standard stimulus (a goat bleats) - non-target the measured participant should not respond
S2 – a dog barks - target – should be followed by the R2 marker
S3 – a cat mews - target - should be followed by the R2 marker
S4 – a cat barks - target - should be followed by the R3 marker
S5 – a dog mews - target - should be followed by the R3 marker

1.1 (page 1): It is not clear why this task is appropriate for understanding the deficits affecting children with DCD. At least one statement regarding deficits in multi-sensory integration and movement time deficits in children with DCD should be included in the purpose statement.

The following text was added:
“ Since standard motors test are relatively time-consuming and physically demanding, there is an open question whether this disorder can be diagnosed using other techniques, such as electroencephalography (EEG) or event-related potentials (ERP). Different studies have been published that investigate link between EEG and DCD. For example,

in~\cite{deCastelnau2008230}, the authors suggest that spectral coherence of certain brain rhythms between different brain regions occurs in children with DCD.

It has been demonstrated that children with DCD have a limited ability to distinguish size, angles, area, and shape compared to children with normal development. Visuospatial processing disorders can be studied using the ERP-based protocol. Furthermore, the high comorbidity~\cite{auditoryERPChildren} between Attention Deficit Hyperactivity Disorder (ADHD) and DCD suggests a possibility of a common developmental anomaly of both disorders. Studies of ERP (in~\cite{pmid8290670} and in~\cite{pmid8879473}) confirmed an attention deficit for both visual and auditory stimuli in children with ADHD. Therefore, given the expected common anomaly in ADHD and DCD, children with DCD should have not only visuospatial attention deficit but also an auditory attention disorder.~\cite{auditoryERPChildren}
“

3) 1.2.5 (page 2): Please include a table with participant details. Given the small sample size and the inherent variability of pediatric data, it would be worthwhile to include this information for ALL participants (and not just the children with DCD or suspected DCD). This table should include: age, sex, MABC raw and percentile scores for the total score and component scores (manual dexterity, ball skills, and balance scores), the degree of hearing impairment, the auditory threshold levels, and whether the child had vision correction.

The table was added. Only component scores of the MABC test (manual dexterity, ball skills, and balance scores) were not included in the table because of the limited space. However, they can be found in the metadata.

1) 1.2 .1 (page 1): Please include the frequency range of these data (i.e., are any data filters applied to the raw data?).

Added into “Recording Hardware”
The raw signal was filtered using an analogue band-pass filter with the cut-off frequencies of 0.1 and 250 Hz.

2) 1.2.4 (page 2): Please include the inter-stimulus interval and jitter in the stimulus presentation.

A figure with the timeline of the experiment has been inserted.

3) 1.2.4 (page 2): Please include the total testing time for each run.

Added into “Stimulation protocol”
Given the number of stimuli and ISI, the total testing time for each run was approximately 6 - 7 minutes.

4) 1.2.5 (page 3): Please state that MABC-2 was used to determine motor abilities and provide the cut-off percentile scores for each group.

Now the manuscript mentiones that MABC-2 was used in our experiment. Cut-off values are mentioned in the Participant chapter.

5) 1.2.7 (page 3): Please include the test date in the metadata so that the age at testing can be calculated.

The dates were corrected.

6) 1.2.7 (page 3): Any confidential participant information from the metadata (name, email) should be removed. It would be useful to also include the MABC total score or MABC total percentile, level of hearing impairment, and visual correction would be useful to add to the metadata.

In the current version of the EEG/ERP portal, names and e-mails of the participants are hidden. . The information in the article saying that the non-anonymized data are available, was incorrect.

7) Figure 1 (page 6): Rather than plotting data with respect to the participant IDs it would be useful to:

a. Plot the individuals by age, since younger children often have more blink artifacts.

b. Color-code the individuals in each group because one would expect the children with DCD to have more eye-blink artifacts.

The figure was updated.
