# Supplementary material for: Developmental coordination disorder in children – experimental work and data annotation
Source: Gigascience. 2017 Feb 24;6(4):1–6. doi: 10.1093/gigascience/gix002 (PMC5530316; doi:10.1093/gigascience/gix002)
Supplement: GIGA-D-16-00094_Revision_2.pdf [file gix002_GIGA-D-16-00094_Revision_2.pdf]

Manuscript Click here to download Manuscript children-dcd.pdf

RESEARCH

Developmental coordination disorder in children - experimental work and data annotation

Lukas Vareka[1*], Petr Bruha[1]
, Roman Moucek[1], Pavel Mautner[1], Ladislav Cepicka[1] and Irena Holeckova[2]

*Correspondence:
lvareka@kiv.zcu.cz
[1]University of West Bohemia,
Univerzitni 8, 306 14, Plzen,
Czech Republic
Full list of author information is available at the end of the article

Abstract

Background: Developmental coordination disorder (DCD) is described as a motor skill disorder characterized by a marked impairment in the development of motor coordination abilities that significantly interferes with performance of daily activities and/or academic achievement. Since some electrophysiological studies suggest differences between children with/without motor development problems, we prepared an experimental protocol and performed electrophysiological experiments with the aim to make a step towards a possible diagnosis of this disorder using the event-related potentials (ERP) technique. The second aim is to properly annotate the obtained raw data with relevant metadata and promote their long term sustainability.

Findings: The data from 32 school children (16 with possible DCD and 16 in the control group) were collected. Each dataset contains raw EEG data in the BrainVision format and provides sufficient metadata (such as age, gender, results of the motor test, and hearing thresholds) to allow other researchers to perform analysis. For each experiment, the percentage of ERP trials damaged by blinking artifacts was estimated. Furthermore, ERP trials were averaged across different participants and conditions, and the resulting plots are included in the manuscript. This should help researchers to estimate the usability of individual datasets for analysis.

Conclusions: The aim of the whole project is to find out if it is possible to make any conclusions about DCD from EEG data obtained. For the purpose of further analysis, the data were collected and annotated respecting the current outcomes of INCF Program on Standards for Data Sharing, Task Force on Electrophysiology and the group developing the Ontology for Experimental Neurophysiology (OEN). The data with metadata are stored in the EEG/ERP Portal.

Keywords: developmental coordination disorder; event-related potentials; visual and audio stimulation; electroencephalography; reaction time

1 Data description

1.1 Theoretical background and purpose of the study

The degree of motor development is usually assessed through clinical tests such a Movement Assessment Battery for Children (MABC-2) [1]. There is an open question whether this disorder can be also diagnosed using other techniques, such as electroencephalography (EEG) or event-related potentials (ERP). EPRs were primarily used as an alternative to measurements of the speed and accuracy of motor responses in paradigms with discrete stimuli and responses [2] and their general advantages when compared to behavioural measures seem worth to be investigated

also in this case. There are two main advantages of ERP technique over behavioral measures. An online measure of stimuli processing can be provided even when there is no behavioral response. The second advantage is that they can provide a continuous measure of processing between a stimulus and a response, making it possible possible to determine which stage or stages of processing are affected by a specific experimental manipulation [2].

Different studies have been published that investigate link between EEG and DCD. For example, in [3], the authors suggest that spectral coherence of certain brain rhythms between different brain regions occurs in children with DCD. It has been demonstrated that children with DCD have a limited ability to distinguish size, angles, area, and shape compared to children with normal development. Visuospatial processing disorders can be studied using the ERP-based protocol. Furthermore, the high comorbidity [4] between Attention Deficit Hyperactivity Disorder (ADHD) and DCD suggests a possibility of a common developmental anomaly of both disorders. Studies of ERP (in [5] and in [6]) confirmed an attention deficit for both visual and auditory stimuli in children with ADHD. Therefore, given the expected common anomaly in ADHD and DCD, children with DCD should have not only visuospatial attention deficit but also an auditory attention disorder. [4]. Our objective was to design and perform event-related potential experiments that can potentially benefit from general advantages of this technique in comparison with traditional behavioral techniques for DCD diagnosis. Although traditional behavioral techniques are fast and relatively inexpensive, EEG, for example, does not need to rely on physical exercise itself and can be used if exercise is currently not possible from medical reasons. Furthermore, EEG can contribute to our understanding about causes of DCD and potential comorbidities. In the long term we would like to influence EEG by some special training (e.g. neurofeedback) and observe if such a training can also influence severity of DCD.

1.2 Participants

The tested subjects were 32 children of younger school age (21 males, 11 females, aged 7-10 years) from a primary school for children with impaired hearing in Pilsen. They were preliminary divided into three groups based on the level of their developmental coordination disorder identified by the MABC-2 motor test [1]. The test evaluates motor performance on three main components: manual dexterity, aiming and catching and balance. The decision was based on the total test score (also referred to as "sum SS") according to a simple Traffic Light system that was proposed in [1]. Children with any score above 67 were in the green zone (no movement difficulty detected). The children that scored between 57 and 67 inclusive were in the yellow zone (at risk of having a movement difficulty). Finally, scores ≤ 56 denoted significant movement difficulty. However, because of a relatively small number of children in the yellow zone, for the purposes of further validation, we decided to merge the yellow zone and the red zone to achieve a group of children with or at risk of DCD. In summary, using the motor test, 16 children were at risk or suffering from DCD (4 out of them were previously in the yellow zone), and 16 were without movement difficulties. All children were right-handed, four children had corrected myopia. Most children suffered from hearing impairment. The level of hearing impairment was assessed using a hearing threshold test. The informed consent was

signed by their legal guardians. All participants with some of the important metadata are listed in Table 1.

Table 1 List of all measured participants. Some of the most important metadata are included (ADD - Attention Deficit Disorder, MBD - Minimal Brain Dysfunction, AS - Asperger Syndrome, ADHD - Attention Deficit Hyperactivity Disorder, DG - Dysgraphia, DO - Dysorthography, DP - Dysphasia, DL - Dyslexia, DLA - Dyslalia, HT - Hearing threshold, MWG - measured without glasses, P - percentile, TS - Total score, VI - Visual impairment). The information about comorbidities were obtained from reports of educational and psychological counselling centres.

ID	Sex	Age	Comorbidities	Myopia (MWG)	HT (db/1kHz)		MABC-2			Eye-blinks (%)
					left	right	TS	SS	P	
276	F	8y 7m	no	no	-5	5	77	9	37	50.4
277	F	7y 6m	ADD	no	-5	5	72	8	25	27.8
278	F	9y 1m	MBD	no	0	0	55	5	5	37
280	F	10y 0m	ADD	no	5	5	55	5	5	44.8
281	M	8y 4m	no	no	20	20	74	9	37	37.5
282	F	9y 11m	MBD	yes (MWG)	25	25	73	9	37	43.3
283	M	8y 4m	ADHD	yes	0	-5	54	5	5	57.5
284	M	8y 1m	AS	no	5	5	61	6	9	40.7
285	M	9y 0m	no	no	20	20	65	7	16	58
286	M	8y 10m	no	no	15	15	88	12	75	38.9
287	M	10y 0m	ADHD	no	5	20	54	5	5	18.2
289	M	8y 3m	DG, DO, DP	no	5	5	43	3	1	43
290	M	8y 7m	DL	yes (MWG)	10	0	54	5	5	29.4
291	M	8y 0m	DP	yes	5	10	85	11	63	37
292	M	7y 5m	DP	no	20	25	70	8	25	25.4
293	F	7y 0m	no	no	20	20	39	3	1	0
294	M	7y 2m	DP	no	5	0	73	9	37	31
295	M	7y 11m	ADD, DP	no	20	20	59	6	9	26.7
296	M	7y 7m	ADHD	no	0	-5	47	4	2	14
795	M	9y 11m	no	no	5	5	77	9	37	33.2
796	M	9y 6m	DLA	no	5	10	56	5	5	66
797	M	9y 9m	no	no	5	0	42	3	1	42.5
798	M	7y 2m	no	no	15	0	80	10	50	40.7
799	M	8y 1m	no	no	5	0	54	5	5	62.9
800	F	7y 7m	no	no	5	5	68	8	25	65.4
801	M	8y 9m	no	no	0	0	63	7	16	57.6
802	F	7y 9m	no	no	20	25	49	2	4	53.5
803	M	7y 3m	ADHD	no	15	5	71	8	25	67.5
804	M	9y 2m	no	no	5	0	93	14	91	67.7
805	F	7y 4m	no	no	20	20	75	9	37	60.9
806	F	8y 1m	no	no	10	10	85	11	63	39.6
807	F	8y 3m	no	no	5	5	97	15	95	47

1.3 Experimental Procedure

The following experimental procedure was applied:

- Each participant was acquainted with the course of the experiment and answered questions concerning his/her health.
- Each participant was given the headphones. The participant was taken to a soundproof and electrically shielded cabin. The hearing threshold for each ear was evaluated. The volume of auditory stimulation was calculated as follows: for each ear, the volume was set to be 50 dB higher than the hearing threshold. However, the volume never exceeded 75 dB.
- Each participant was given a standard 10-20 system EEG cap and headphones. 19 electrodes were used as depicted in Fig. 1. The participant was taken to a soundproof and electrically shielded cabin; the reference electrode was placed at the root of his/her nose.
- The participant was told to watch the pictures on the screen, to listen to the sounds, and to respond to stimuli as described in Section 1.4.4.

- The cabin was closed and both the data recording and stimulation started. Fig. 2 shows a participant during the experiment.
- After the experiment had finished, the recorded data and collected metadata were uploaded to the EEG/ERP Portal. [7]

[scale=0.2]figures/TheinternationalStandard10-2019electrode.pdf

Figure 1 The locations of the electrodes attached in the 10-20 system.

[scale=0.8]figures/photo_exp.pdf

Figure 2 A participant during the experiment.

1.4 EEG data recording

1.4.1 Recording Hardware

The standard 10-20 system EEG cap made by Electro-Cap International was used for the experiment. The EEG cap contained 19 electrodes. The BrainAmp DC amplifier was used with the sampling frequency set to 1 kHz. The raw signal was filtered using an analogue band-pass filter with the cut-off frequencies of 0.1 and 250 Hz. There were two buttons placed at the armrests of the chair for measuring reactions of participants (also depicted in Fig. 2).

1.4.2 Recording Software

The BrainVision Recorder 1.2 [8] was used for recording and storing the EEG/ERP data in the BrainVision format. The impedance threshold was set to 10 $k\Omega$; the real impedances for each experiment were stored in vhdr files. The Presentation software [9], version 16.3 made by the Neurobehavioral Systems was used for stimulation.

1.4.3 Environment

All experiments were performed in a sound and electrically shielded booth placed in an electrophysiology lab. EEG/ERP activity was recorded using the standard 10-20 international system with the reference electrode placed at the root of the nose.

1.4.4 Stimulation protocol

The experimental protocol was based on multimodal stimulation, i.e. a combination of auditory and visual stimulation. The visual stimuli were represented by pictures of animals. The corresponding auditory stimuli were represented by sounds of the animals that occurred in synchronization with the visual stimuli. One of the pictures (a goat), occurring with the probability of 70%, was always associated with the correct sound, and was the standard (non-target) stimulus. In rare stimuli, the sounds might be incorrectly associated with the animals. The rare stimuli included: barking dog (15%), meowing cat (5%), meowing dog (5%), and barking cat (5%). 600 stimuli were used in total during the experimental session. Each experimental

session was divided into two experimental runs, each containing 300 stimuli. During the experimental session, participants were asked to reply to each target stimulus (dog or cat sound) by pressing one button for sounds of barking dog or meowing cat, and the other button for sounds of barking cat or meowing dog.

Inter-stimulus interval (ISI) was 1200 ms, response interval between 200 ms and 1000 ms after each stimulus, and trial length was set to 1200 ms. Given the number of stimuli and ISI, the total testing time for each run was approximately 6 - 7 minutes. Fig. 3 depicts the course of the experiment.

[scale=0.8]figures/timeline.pdf

Figure 3 Course of the experiment. Each stimulation marker was associated with 700 ms of sound and visual stimulation. Subsequently, 500 ms without stimulation followed. Therefore, inter-stimulus interval was 1200 ms. The responses of the subjects were considered on time between 200 ms and 1000 ms after each stimulus.

1.4.5 Data and metadata

The collected data and metadata were stored in the EEG/ERP Portal. The metadata include, for example:

1. weather conditions
2. used hardware
3. start time and end time of the experiment
4. temperature in the laboratory
5. used stimulation protocol (scenario title, description, length, source file)
6. information about the participant (gender, age, laterality, diseases, etc.)

In addition, experiment-specific metadata about motoric percentiles [10] and hearing thresholds were stored in separate text files along with the datasets.

Finally, for each experiment, important information about behavioral responses of the participants, including reaction times to each stimulus and average reaction times, are stored in the LOG_multimod folders. In the same folder, there also is a file describing the format of these metadata.

1.5 Data Validation

First, epochs were averaged for both groups (with and without DCD). The results for the Pz channel are depicted in Fig. 4.

[scale=0.7]figures/allcmp19.pdf

Figure 4 Averages for each participant and each stimulus marker are shown. Figures are divided into two groups based on the condition of the participants (i.e. with DCD / without DCD). Grand averages for each marker are depicted in a red bold line. The Pz channel was averaged. Markers used are in detail explained in the attached metadata. S1 – standard stimulus (a goat bleats), S2 - target stimulus (a dog barks), S3 – target stimulus (a cat mews), S4 – target stimulus (a cat barks), S5 – target stimulus (a dog mews).

To evaluate the quality of the data for different subjects, percentage of eye-blinking artifacts was estimated using visual inspection. The results are depicted in Fig. 5. Although eye blinks cause significant disruptions in the EEG signal, they can be partially corrected using Independent Component Analysis (ICA). Therefore, to be

able to analyze EEG without excessive data loss even for subjects that blink a lot, ICA e.g. from EEGLAB or BrainVision should be performed.

[scale=0.8]figures/artifactsAge.pdf

Figure 5 Percentage of eye-blinking artifacts for each age group also divided by the condition of the participants (i.e. with DCD / without DCD).

2 Availability and requirements

To download the data described in this article, the following project is available:

- Project name: EEG/ERP Portal [7]
  Project home page: http://eegdatabase.kiv.zcu.cz
  Operating system(s): Platform independent
  Programming language: Java
  Other requirements: tested in Internet Explorer 10, 11, Mozilla Firefox 29.0.1, Google Chrome
  License: Creative Commons BY-NC-SA 4.0

3 Availability of supporting data

The experimental data and metadata can be downloaded from the EEG/ERP Portal according to the following procedure. Any user has to be registered first. When the registration form is completed, a confirmation e-mail is sent to the user. Then the user is requested to click on the confirmation link contained in the confirmation e-mail. After a successful login a personalized user's homepage including an overview of user's experiments, scenarios, research group memberships, etc. is displayed. In order to see publicly offered experiments and find the package named 'Developmental coordination disorder in children – experimental work and data annotation', the user selects the Experiments section from the main menu appearing at the top of the homepage. When the Experiment section is loaded, the user selects the package 'Developmental coordination disorder in children – experimental work and data annotation', chooses the license under which he/she wants to use the data (Creative Commons BY-NC) and clicks on the 'Add to cart' link (free of charge).

When the package is added into the cart, the user is requested to click on the 'My cart' link at the top of the page. The experiments in the selected package are available under the selected license. When the user finishes the order (by clicking on the 'Create order' button), the download page finally appears (by clicking on the 'Download' link). Then the user confirms his/her selection of the experiments within the package and clicks on the 'Create package' button to create a zip package. Since the data are quite large, the progress bar indicates the portion of the package that has been already created. When the package is created, it can be finally downloaded by clicking on the 'Download' link.

The ordered (purchased) package could be re-downloaded at any time in the Experiment section by clicking on the 'Download' link that appears instead of the 'Add to cart' link within the package.

Abbreviations

DCD: Developmental coordination disorder; EEG: electroencephalography; ERP: event-related potentials; INCF: International Neuroinformatics Coordinating Facility; URL: Uniform Resource Locator.

Competing interests

The authors declare that they have no competing interests.

Author's contributions

IH, LC and PM designed the experiments. PB, PM and LC performed the experiments. LV designed the data validation method and analyzed the data. PB prepared datasets for storing. LV, RM and PB wrote the paper. All authors read and approved the final manuscript.

Acknowledgements

This work was supported by the Grant Agency of the Czech Republic under the grant P407/12/1525.

Author details

[1]University of West Bohemia, Univerzitni 8, 306 14, Plzen, Czech Republic. [2]University Hospital Plzen, Alej Svobody 80, 304 60, Plzen, Czech Republic.

References

1. Henderson, S.E., Sugden, D.D.A.., Barnett, A.L., Corporation, P.: Movement Assessment Battery for Children-2. London : Harcourt Assessment. Title from Examiner's manual cover (2007)
2. Luck, S.J.: An Introduction to the Event-related Potential Technique. MIT press, ??? (2014)
3. de Castelnau, P., Albaret, J.-M., Chaix, Y., Zanone, P.-G.: A study of {EEG} coherence in {DCD} children during motor synchronization task. Human Movement Science **27**(2), 230–241 (2008). doi:10.1016/j.humov.2008.02.006. Developmental Coordination Disorder
4. Holeckova, I., Cepicka, L., Mautner, P., Stepanek, D., Moucek, R.: Auditory erps in children with developmental coordination disorder. Activitas Nervosa Superior, 37–44 (2014)
5. Winsberg, B.G., Javitt, D.C., Silipo, G.S., Doneshka, P.: Mismatch negativity in hyperactive children: effects of methylphenidate. Psychopharmacol Bull **29**(2), 229–233 (1993)
6. Kemner, C., Verbaten, M.N., Koelega, H.S., Buitelaar, J.K., van der Gaag, R.J., Camfferman, G., van Engeland, H.: Event-related brain potentials in children with attention-deficit and hyperactivity disorder: effects of stimulus deviancy and task relevance in the visual and auditory modality. Biol. Psychiatry **40**(6), 522–534 (1996)
7. Moucek, R., Jezek, P.: EEG/ERP Portal. http://eegdatabase.kiv.zcu.cz/
8. BrainProducts: Brain Vision Recorder. www.brainproducts.com/productdetails.php?id=21
9. NeurobehavioralSystems: Presentation. http://www.neurobs.com/
10. Gueze, R.H., Jongmans, M.J., Schoemaker, M.M., Smits-Engelsman, B.C.: Clinical and research diagnostic criteria for developmental coordination disorder: a review and discussion. Hum Mov Sci **20**(1-2), 7–47 (2001)

Dear reviewers,

thank you for your valuable feedback. Below, you can see our response to your comments.

Best regards,
Lukas Vareka, Petr Bruha, Roman Moucek

Major Compulsory Revision:

1. **Common motor skill assessments that are used in this population, like the MABC-2, BOT-2, or TGMD-2, are relatively fast to administer (~30-45 minutes). Although setting up an EEG cap with 19 electrodes may be very fast, I do not think that the speed alone justifies the use of EEG as a replacement for traditional behavioral assessments. It would be worthwhile to also add the approximate total time (set up + paradigm) and to substantiate the utility of EEG. What information is gained that cannot be gained using behavioral assessments? Moreover, the expense of these behavioral assessments is much reduced compared with most EEG systems (i.e., ~$1000 or less compared with $80,000 for a BrainVision EEG system). As such, the important thing about the use of EEG systems is that they are able to do a better job of discriminating differences or add something unique to our understanding of DCD and the potential comorbidities. These points, the time and cost considerations, could be highlighted in the introduction or discussion.**

This discussion was added into the “Theoretical background and purpose of the study” chapter.

2. Given the comorbidity of DCD and other developmental/learning disabilities (e.g., Autism Spectrum Disorder, ADHD, developmental dyslexia), it would be worthwhile to add in the metadata or participant information the existence of comorbidities and how these comorbidities were assessed (e.g., parent report, neuropsychological/educational tests, physician diagnosis). Without this information: A) it would be difficult to use this data repository to reliably combine data from different research groups and B) it would be impossible to use data-driven discrimination procedures to predict group membership (e.g., control, probably DCD, DCD, DCD+ADHD, DCD+ASD, etc.). Based on the introduction, I believe that the ultimate goal would be to address both A and B.

The information about comorbidities have been collected from reports of educational and psychological counselling centres and are now included in the metadata.

3. The total scores from the MABC-2 are not typically reported. The percentiles

are more meaningful because the red zone is characterized as at or below the 5th percentile, the amber zone is between the 6th and 15th percentile, and the green is at or above the 15th percentile. As such, the description of the MABC under 1.2 Participants should be changed to reflect the language in the MABC-2 manual (see above or page 176 of the manual). Also, the table provides "Sum SS" which should be changed to "Total Score".

We were following the ample system mentioned in:

Henderson, S.E., Sugden, D.D.A.., Barnett, A.L., Corporation, P.: Movement Assessment Battery for Children-2.
London : Harcourt Assessment. Title from Examiner's manual cover (2007).

On page 176 there is a table::

Movement ABC-2 Test 'Traffic Light' System

Some users might find a simple 'Traffic Light' system useful when explaining a child's Test score to parents, teachers or learning support staff. This system is based on percentiles only. The definition of each zone is as follows:

Table 3b: Traffic Light system for Total Test Score

Child's score	Total Test Score	Percentile range	Description
Red zone	up to and including 56	at or below the 5th percentile	denotes a significant movement difficulty
Amber zone	between 57 and 67 inclusive	between the 5th and 15th percentile inclusive	suggests the child is 'at risk' of having a movement difficulty; monitoring required
Green zone	any score above 67	above the 15th percentile	no movement difficulty detected

Note: as there is no direct correspondence between the 15th percentile and a standard score, in this instance the 16th percentile point has been taken as the upper end of the 'amber' range, equivalent to a standard score of 7.

In the Movement ABC-2 Test, **poor** performance is represented by **low** standard scores. In contrast, in the Movement ABC-2 Checklist, **high** scores represent **poor** performance. In this case, the red zone represents scores at or above the 95th percentile, the amber zone between the 85th and the 94th percentile inclusive, and the green zone any score below the 85th percentile. (See page 96 for the equivalent table for the Checklist).

We were following the Total test score in the table 3b to obtain two equally sized groups of test subjects. It is definitely simple to switch to percentiles, however, two subjects with the percentile of 16 would fall into the green zone. Now, they are in the amber zone (having the total test score of 63 and 65). Such a strange overlap would mean that with percentiles, we would have one group of 14 and one group of 18 in the situation when getting more subjects is very complicated and nearly impossible.

Minor Compulsory Revisions:
1. In the table, age in months should be used instead of age in years.

The information about ages were updated in the metadata.

2. Were the participants with visual impairments wearing corrective lenses during the task?

The information whether the participants were wearing corrective lenses is mentioned in the metadata.

3. Blink artifacts are easily removed without data loss with algorithms like independent components analysis. These algorithms are now included in EEGlab or BrainVision Analyzer 2 and are very useful for young children and those that tend to blink a lot. Given a reduction in data loss, the paradigm may be able to be shortened. The authors could provide this as a suggestion.

Independent component analysis is now mentioned in the manuscript. We did not perform ICA because we wanted to provide raw data.
