# Supplementary material for: Developmental coordination disorder in children – experimental work and data annotation
Source: Gigascience. 2017 Feb 24;6(4):1–6. doi: 10.1093/gigascience/gix002 (PMC5530316; doi:10.1093/gigascience/gix002)

Manuscript Click here to download Manuscript children-dcd.pdf

RESEARCH

Developmental coordination disorder in children - experimental work and data annotation

Lukas Vareka[1*], Petr Bruha[1]
, Roman Moucek[1], Pavel Mautner[1], Ladislav Cepicka[1] and Irena Holeckova[2]

*Correspondence:
lvareka@kiv.zcu.cz
[1]University of West Bohemia, Univerzitni 8, 306 14, Plzen, Czech Republic
Full list of author information is available at the end of the article

Abstract

Background: Developmental coordination disorder (DCD) is described as a motor skill disorder characterized by a marked impairment in the development of motor coordination abilities that significantly interferes with performance of daily activities and/or academic achievement. Since some electrophysiological studies suggest differences between children with/without motor development problems, we prepared an experimental protocol and performed electrophysiological experiments with the aim to make a step towards a possible diagnosis of this disorder using the event-related potentials (ERP) technique. The second aim is to properly annotate the obtained raw data with relevant metadata and promote their long term sustainability.

Findings: The data from 32 school children (16 with possible DCD and 16 in the control group) were collected. Each dataset contains raw EEG data in the BrainVision format and provides sufficient metadata (such as age, gender, results of the motor test, and hearing thresholds) to allow other researchers to perform analysis. For each experiment, the percentage of ERP trials damaged by blinking artifacts was estimated. Furthermore, ERP trials were averaged across different participants and conditions, and the resulting plots are included in the manuscript. This should help researchers to estimate the usability of individual datasets for analysis.

Conclusions: The aim of the whole project is to find out if it is possible to make any conclusions about DCD from EEG data obtained. For the purpose of further analysis, the data were collected and annotated respecting the current outcomes of INCF Program on Standards for Data Sharing, Task Force on Electrophysiology and the group developing the Ontology for Experimental Neurophysiology (OEN). The data with metadata are stored in the EEG/ERP Portal.

Keywords: developmental coordination disorder; event-related potentials; visual and audio stimulation; electroencephalography; reaction time

1 Data description

1.1 Theoretical background and purpose of the study

The degree of motor development is usually assessed through clinical tests such a Movement Assessment Battery for Children (MABC-2) [1]. There is an open question whether this disorder can be also diagnosed using other techniques, such as electroencephalography (EEG) or event-related potentials (ERP). EPRs were primarily used as an alternative to measurements of the speed and accuracy of motor responses in paradigms with discrete stimuli and responses [2] and their general advantages when compared to behavioural measures seem worth to be investigated

also in this case. There are two main advantages of ERP technique over behavioral measures. An online measure of stimuli processing can be provided even when there is no behavioral response. The second advantage is that they can provide a continuous measure of processing between a stimulus and a response, making it possible possible to determine which stage or stages of processing are affected by a specific experimental manipulation [2].

Different studies have been published that investigate link between EEG and DCD. For example, in [3], the authors suggest that spectral coherence of certain brain rhythms between different brain regions occurs in children with DCD. It has been demonstrated that children with DCD have a limited ability to distinguish size, angles, area, and shape compared to children with normal development. Visuospatial processing disorders can be studied using the ERP-based protocol. Furthermore, the high comorbidity [4] between Attention Deficit Hyperactivity Disorder (ADHD) and DCD suggests a possibility of a common developmental anomaly of both disorders. Studies of ERP (in [5] and in [6]) confirmed an attention deficit for both visual and auditory stimuli in children with ADHD. Therefore, given the expected common anomaly in ADHD and DCD, children with DCD should have not only visuospatial attention deficit but also an auditory attention disorder. [4]. Our objective was to design and perform event-related potential experiments that can potentially benefit from general advantages of this technique in comparison with traditional behavioral techniques for DCD diagnosis. Although traditional behavioral techniques are fast and relatively inexpensive, EEG, for example, does not need to rely on physical exercise itself and can be used if exercise is currently not possible from medical reasons. Furthermore, EEG can contribute to our understanding about causes of DCD and potential comorbidities. In the long term we would like to influence EEG by some special training (e.g. neurofeedback) and observe if such a training can also influence severity of DCD.

1.2 Participants

The tested subjects were 32 children of younger school age (21 males, 11 females, aged 7-10 years) from a primary school for children with impaired hearing in Pilsen. They were preliminary divided into three groups based on the level of their developmental coordination disorder identified by the MABC-2 motor test [1]. The test evaluates motor performance on three main components: manual dexterity, aiming and catching and balance. The decision was based on the total test score (also referred to as "sum SS") according to a simple Traffic Light system that was proposed in [1]. Children with any score above 67 were in the green zone (no movement difficulty detected). The children that scored between 57 and 67 inclusive were in the yellow zone (at risk of having a movement difficulty). Finally, scores ≤ 56 denoted significant movement difficulty. However, because of a relatively small number of children in the yellow zone, for the purposes of further validation, we decided to merge the yellow zone and the red zone to achieve a group of children with or at risk of DCD. In summary, using the motor test, 16 children were at risk or suffering from DCD (4 out of them were previously in the yellow zone), and 16 were without movement difficulties. All children were right-handed, four children had corrected myopia. Most children suffered from hearing impairment. The level of hearing impairment was assessed using a hearing threshold test. The informed consent was

signed by their legal guardians. All participants with some of the important metadata are listed in Table 1.

Table 1 List of all measured participants. Some of the most important metadata are included (ADD - Attention Deficit Disorder, MBD - Minimal Brain Dysfunction, AS - Asperger Syndrome, ADHD - Attention Deficit Hyperactivity Disorder, DG - Dysgraphia, DO - Dysorthography, DP - Dysphasia, DL - Dyslexia, DLA - Dyslalia, HT - Hearing threshold, MWG - measured without glasses, P - percentile, TS - Total score, VI - Visual impairment). The information about comorbidities were obtained from reports of educational and psychological counselling centres.

ID	Sex	Age	Comorbidities	Myopia (MWG)	HT (db/1kHz)		MABC-2			Eye-blinks (%)
					left	right	TS	SS	P	
276	F	8y 7m	no	no	-5	5	77	9	37	50.4
277	F	7y 6m	ADD	no	-5	5	72	8	25	27.8
278	F	9y 1m	MBD	no	0	0	55	5	5	37
280	F	10y 0m	ADD	no	5	5	55	5	5	44.8
281	M	8y 4m	no	no	20	20	74	9	37	37.5
282	F	9y 11m	MBD	yes (MWG)	25	25	73	9	37	43.3
283	M	8y 4m	ADHD	yes	0	-5	54	5	5	57.5
284	M	8y 1m	AS	no	5	5	61	6	9	40.7
285	M	9y 0m	no	no	20	20	65	7	16	58
286	M	8y 10m	no	no	15	15	88	12	75	38.9
287	M	10y 0m	ADHD	no	5	20	54	5	5	18.2
289	M	8y 3m	DG, DO, DP	no	5	5	43	3	1	43
290	M	8y 7m	DL	yes (MWG)	10	0	54	5	5	29.4
291	M	8y 0m	DP	yes	5	10	85	11	63	37
292	M	7y 5m	DP	no	20	25	70	8	25	25.4
293	F	7y 0m	no	no	20	20	39	3	1	0
294	M	7y 2m	DP	no	5	0	73	9	37	31
295	M	7y 11m	ADD, DP	no	20	20	59	6	9	26.7
296	M	7y 7m	ADHD	no	0	-5	47	4	2	14
795	M	9y 11m	no	no	5	5	77	9	37	33.2
796	M	9y 6m	DLA	no	5	10	56	5	5	66
797	M	9y 9m	no	no	5	0	42	3	1	42.5
798	M	7y 2m	no	no	15	0	80	10	50	40.7
799	M	8y 1m	no	no	5	0	54	5	5	62.9
800	F	7y 7m	no	no	5	5	68	8	25	65.4
801	M	8y 9m	no	no	0	0	63	7	16	57.6
802	F	7y 9m	no	no	20	25	49	2	4	53.5
803	M	7y 3m	ADHD	no	15	5	71	8	25	67.5
804	M	9y 2m	no	no	5	0	93	14	91	67.7
805	F	7y 4m	no	no	20	20	75	9	37	60.9
806	F	8y 1m	no	no	10	10	85	11	63	39.6
807	F	8y 3m	no	no	5	5	97	15	95	47

1.3 Experimental Procedure

The following experimental procedure was applied:

- Each participant was acquainted with the course of the experiment and answered questions concerning his/her health.
- Each participant was given the headphones. The participant was taken to a soundproof and electrically shielded cabin. The hearing threshold for each ear was evaluated. The volume of auditory stimulation was calculated as follows: for each ear, the volume was set to be 50 dB higher than the hearing threshold. However, the volume never exceeded 75 dB.
- Each participant was given a standard 10-20 system EEG cap and headphones. 19 electrodes were used as depicted in Fig. 1. The participant was taken to a soundproof and electrically shielded cabin; the reference electrode was placed at the root of his/her nose.
- The participant was told to watch the pictures on the screen, to listen to the sounds, and to respond to stimuli as described in Section 1.4.4.

- The cabin was closed and both the data recording and stimulation started. Fig. 2 shows a participant during the experiment.
- After the experiment had finished, the recorded data and collected metadata were uploaded to the EEG/ERP Portal. [7]

[scale=0.2]figures/TheinternationalStandard10-2019electrode.pdf

Figure 1 The locations of the electrodes attached in the 10-20 system.

[scale=0.8]figures/photo_exp.pdf

Figure 2 A participant during the experiment.

1.4 EEG data recording

1.4.1 Recording Hardware

The standard 10-20 system EEG cap made by Electro-Cap International was used for the experiment. The EEG cap contained 19 electrodes. The BrainAmp DC amplifier was used with the sampling frequency set to 1 kHz. The raw signal was filtered using an analogue band-pass filter with the cut-off frequencies of 0.1 and 250 Hz. There were two buttons placed at the armrests of the chair for measuring reactions of participants (also depicted in Fig. 2).

1.4.2 Recording Software

The BrainVision Recorder 1.2 [8] was used for recording and storing the EEG/ERP data in the BrainVision format. The impedance threshold was set to 10 $k\Omega$; the real impedances for each experiment were stored in vhdr files. The Presentation software [9], version 16.3 made by the Neurobehavioral Systems was used for stimulation.

1.4.3 Environment

All experiments were performed in a sound and electrically shielded booth placed in an electrophysiology lab. EEG/ERP activity was recorded using the standard 10-20 international system with the reference electrode placed at the root of the nose.

1.4.4 Stimulation protocol

The experimental protocol was based on multimodal stimulation, i.e. a combination of auditory and visual stimulation. The visual stimuli were represented by pictures of animals. The corresponding auditory stimuli were represented by sounds of the animals that occurred in synchronization with the visual stimuli. One of the pictures (a goat), occurring with the probability of 70%, was always associated with the correct sound, and was the standard (non-target) stimulus. In rare stimuli, the sounds might be incorrectly associated with the animals. The rare stimuli included: barking dog (15%), meowing cat (5%), meowing dog (5%), and barking cat (5%). 600 stimuli were used in total during the experimental session. Each experimental

session was divided into two experimental runs, each containing 300 stimuli. During the experimental session, participants were asked to reply to each target stimulus (dog or cat sound) by pressing one button for sounds of barking dog or meowing cat, and the other button for sounds of barking cat or meowing dog.

Inter-stimulus interval (ISI) was 1200 ms, response interval between 200 ms and 1000 ms after each stimulus, and trial length was set to 1200 ms. Given the number of stimuli and ISI, the total testing time for each run was approximately 6 - 7 minutes. Fig. 3 depicts the course of the experiment.

[scale=0.8]figures/timeline.pdf

Figure 3 Course of the experiment. Each stimulation marker was associated with 700 ms of sound and visual stimulation. Subsequently, 500 ms without stimulation followed. Therefore, inter-stimulus interval was 1200 ms. The responses of the subjects were considered on time between 200 ms and 1000 ms after each stimulus.

1.4.5 Data and metadata

The collected data and metadata were stored in the EEG/ERP Portal. The metadata include, for example:

1 weather conditions
2 used hardware
3 start time and end time of the experiment
4 temperature in the laboratory
5 used stimulation protocol (scenario title, description, length, source file)
6 information about the participant (gender, age, laterality, diseases, etc.)

In addition, experiment-specific metadata about motoric percentiles [10] and hearing thresholds were stored in separate text files along with the datasets.

Finally, for each experiment, important information about behavioral responses of the participants, including reaction times to each stimulus and average reaction times, are stored in the LOG_multimod folders. In the same folder, there also is a file describing the format of these metadata.

1.5 Data Validation

First, epochs were averaged for both groups (with and without DCD). The results for the Pz channel are depicted in Fig. 4.

[scale=0.7]figures/allcmp19.pdf

Figure 4 Averages for each participant and each stimulus marker are shown. Figures are divided into two groups based on the condition of the participants (i.e. with DCD / without DCD). Grand averages for each marker are depicted in a red bold line. The Pz channel was averaged. Markers used are in detail explained in the attached metadata. S1 – standard stimulus (a goat bleats), S2 - target stimulus (a dog barks), S3 – target stimulus (a cat mews), S4 – target stimulus (a cat barks), S5 – target stimulus (a dog mews).

To evaluate the quality of the data for different subjects, percentage of eye-blinking artifacts was estimated using visual inspection. The results are depicted in Fig. 5. Although eye blinks cause significant disruptions in the EEG signal, they can be partially corrected using Independent Component Analysis (ICA). Therefore, to be

able to analyze EEG without excessive data loss even for subjects that blink a lot, ICA e.g. from EEGLAB or BrainVision should be performed.

[scale=0.8]figures/artifactsAge.pdf

Figure 5 Percentage of eye-blinking artifacts for each age group also divided by the condition of the participants (i.e. with DCD / without DCD).

2 Availability of supporting data

Snapshots of the data described here are available under a CC0 waiver from the GigaScience GigaDB repository [11]. The latest experimental data and metadata can also be downloaded from the EEG/ERP Portal [7] according to the following procedure. This has been tested in Internet Explorer 10, 11, Mozilla Firefox 29.0.1, and Google Chrome. Any user has to be registered first. When the registration form is completed, a confirmation e-mail is sent to the user. Then the user is requested to click on the confirmation link contained in the confirmation e-mail. After a successful login a personalized user's homepage including an overview of user's experiments, scenarios, research group memberships, etc. is displayed. In order to see publicly offered experiments and find the package named 'Developmental coordination disorder in children – experimental work and data annotation', the user selects the Experiments section from the main menu appearing at the top of the homepage. When the Experiment section is loaded, the user selects the package 'Developmental coordination disorder in children – experimental work and data annotation', chooses the license under which he/she wants to use the data (Creative Commons BY-NC is the default) and clicks on the 'Add to cart' link (free of charge).

When the package is added into the cart, the user is requested to click on the 'My cart' link at the top of the page. The experiments in the selected package are available under the selected license. When the user finishes the order (by clicking on the 'Create order' button), the download page finally appears (by clicking on the 'Download' link). Then the user confirms his/her selection of the experiments within the package and clicks on the 'Create package' button to create a zip package. Since the data are quite large, the progress bar indicates the portion of the package that has been already created. When the package is created, it can be finally downloaded by clicking on the 'Download' link.

The ordered (purchased) package could be re-downloaded at any time in the Experiment section by clicking on the 'Download' link that appears instead of the 'Add to cart' link within the package.

Abbreviations
DCD: Developmental coordination disorder; EEG: electroencephalography; ERP: event-related potentials; INCF: International Neuroinformatics Coordinating Facility; URL: Uniform Resource Locator.

Competing interests
The authors declare that they have no competing interests.

Author's contributions
IH, LC and PM designed the experiments. PB, PM and LC performed the experiments. LV designed the data validation method and analyzed the data. PB prepared datasets for storing. LV, RM and PB wrote the paper. All authors read and approved the final manuscript.

Acknowledgements
This work was supported by the Grant Agency of the Czech Republic under the grant P407/12/1525.

Author details
[1]University of West Bohemia, Univerzitni 8, 306 14, Plzen, Czech Republic. [2]University Hospital Plzen, Alej Svobody 80, 304 60, Plzen, Czech Republic.

References

1. Henderson, S.E., Sugden, D.D.A.., Barnett, A.L., Corporation, P.: Movement Assessment Battery for Children-2. London : Harcourt Assessment. Title from Examiner's manual cover (2007)
2. Luck, S.J.: An Introduction to the Event-related Potential Technique. MIT press, ??? (2014)
3. de Castelnau, P., Albaret, J.-M., Chaix, Y., Zanone, P.-G.: A study of {EEG} coherence in {DCD} children during motor synchronization task. Human Movement Science **27**(2), 230–241 (2008). doi:10.1016/j.humov.2008.02.006. Developmental Coordination Disorder
4. Holeckova, I., Cepicka, L., Mautner, P., Stepanek, D., Moucek, R.: Auditory erps in children with developmental coordination disorder. Activitas Nervosa Superior, 37–44 (2014)
5. Winsberg, B.G., Javitt, D.C., Silipo, G.S., Doneshka, P.: Mismatch negativity in hyperactive children: effects of methylphenidate. Psychopharmacol Bull **29**(2), 229–233 (1993)
6. Kemner, C., Verbaten, M.N., Koelega, H.S., Buitelaar, J.K., van der Gaag, R.J., Camfferman, G., van Engeland, H.: Event-related brain potentials in children with attention-deficit and hyperactivity disorder: effects of stimulus deviancy and task relevance in the visual and auditory modality. Biol. Psychiatry **40**(6), 522–534 (1996)
7. Moucek, R., Jezek, P.: EEG/ERP Portal. http://eegdatabase.kiv.zcu.cz/
8. BrainProducts: Brain Vision Recorder. www.brainproducts.com/productdetails.php?id=21
9. NeurobehavioralSystems: Presentation. http://www.neurobs.com/
10. Gueze, R.H., Jongmans, M.J., Schoemaker, M.M., Smits-Engelsman, B.C.: Clinical and research diagnostic criteria for developmental coordination disorder: a review and discussion. Hum Mov Sci **20**(1-2), 7–47 (2001)
11. Vareka, L., Bruha, P., Moucek, R., Mautner, P., Cepicka, L., Holeckova, I.: Supporting data for 'developmental coordination disorder in children experimental work and data annotation'. GigaScience Database (2016). doi:10.5524/100260

Figure 1

Click here to download Figure 1.pdf

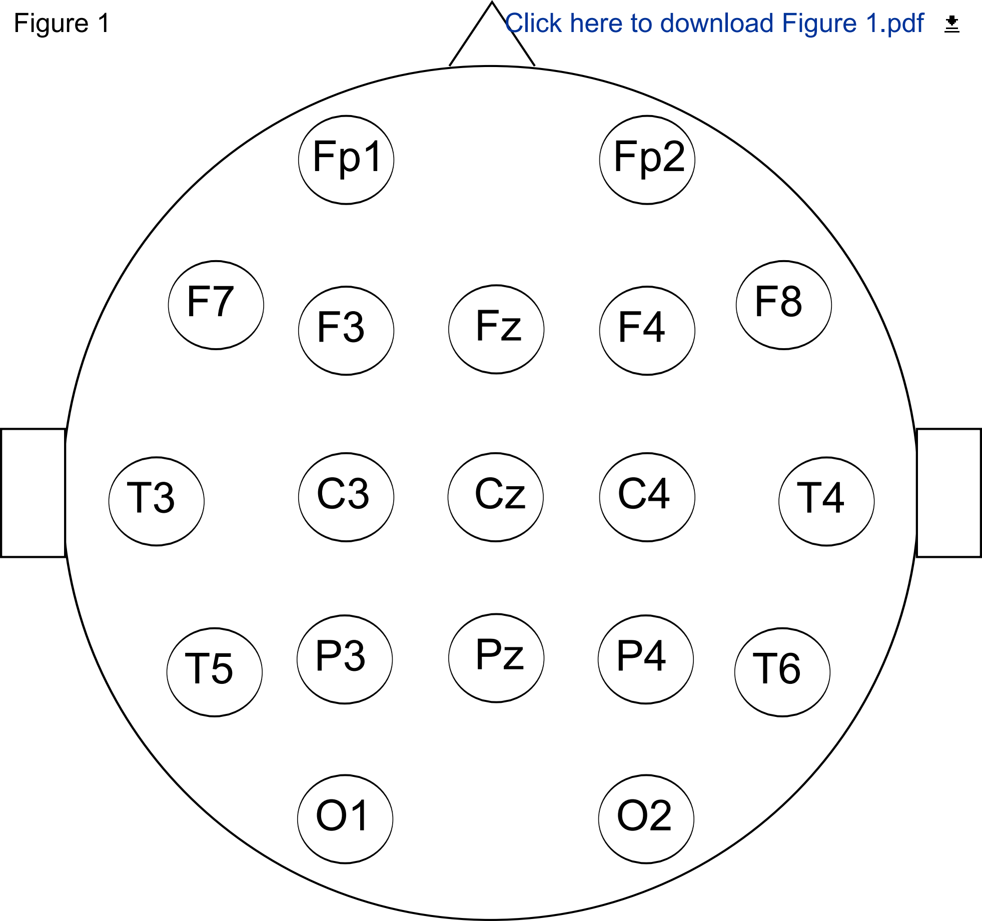

Figure 2

Click here to download Figure Fig2.pdf

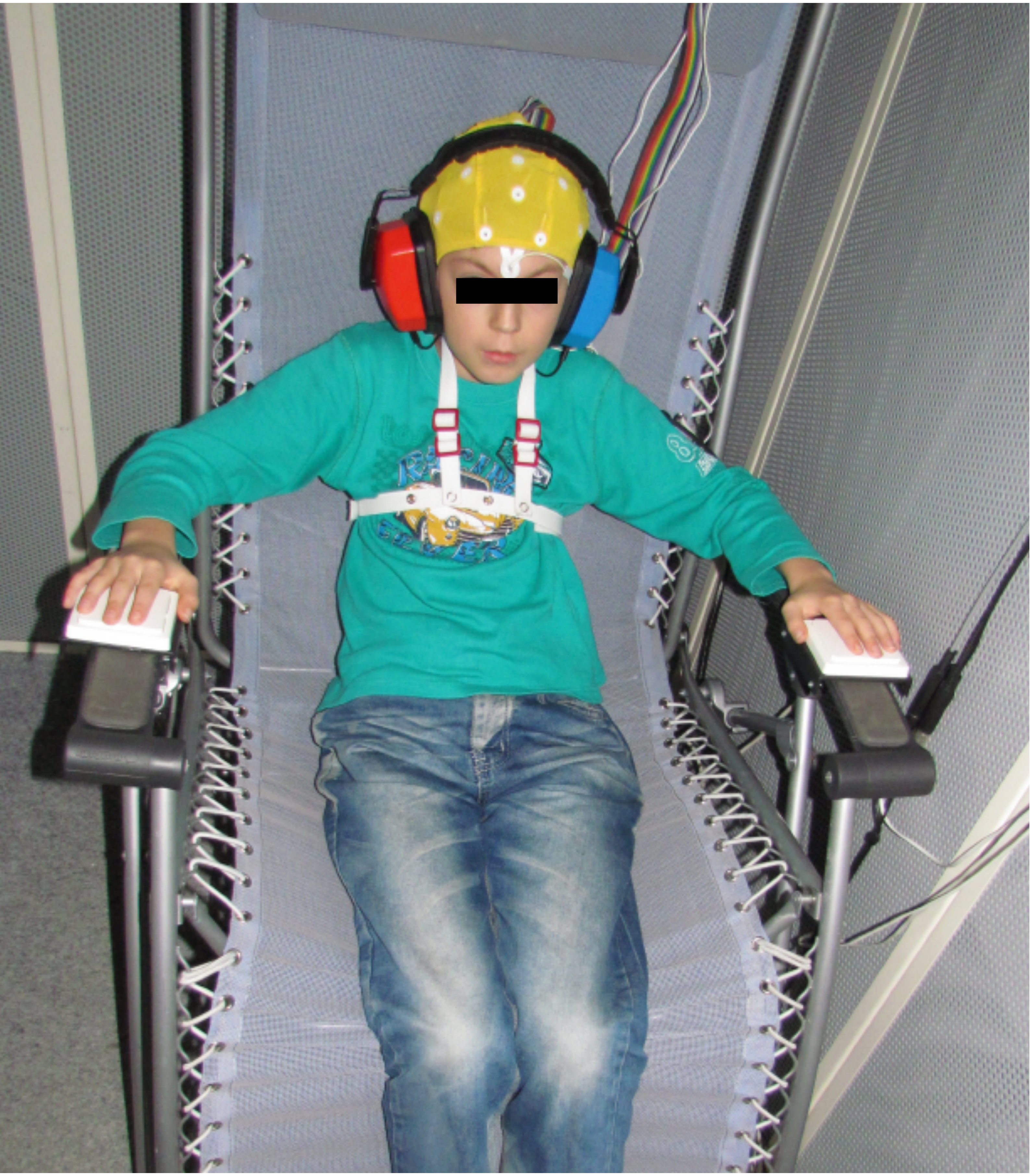

Figure 3

Click here to download Figure 3.pdf

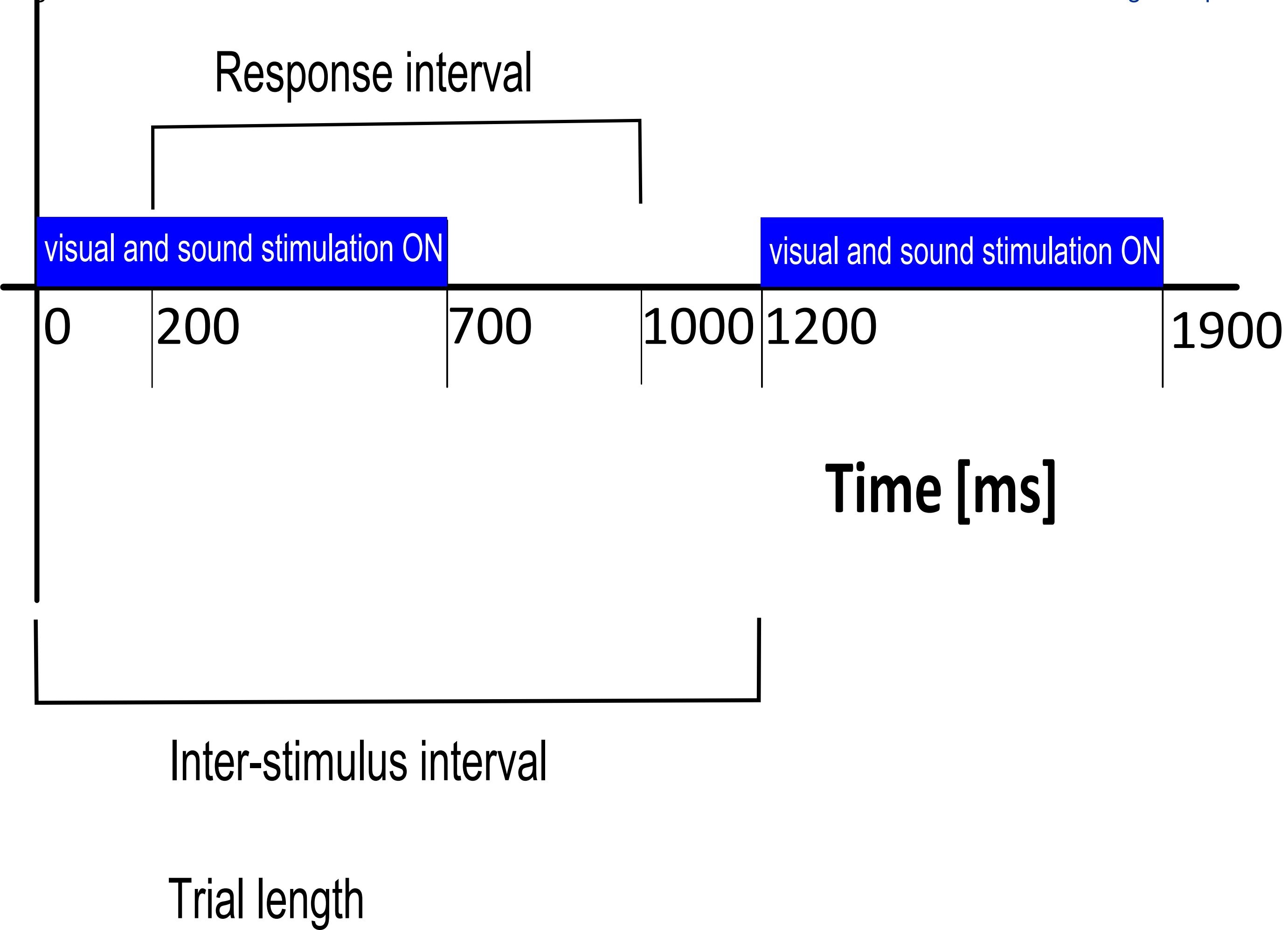

Figure 4

Click here to download Figure 4.pdf

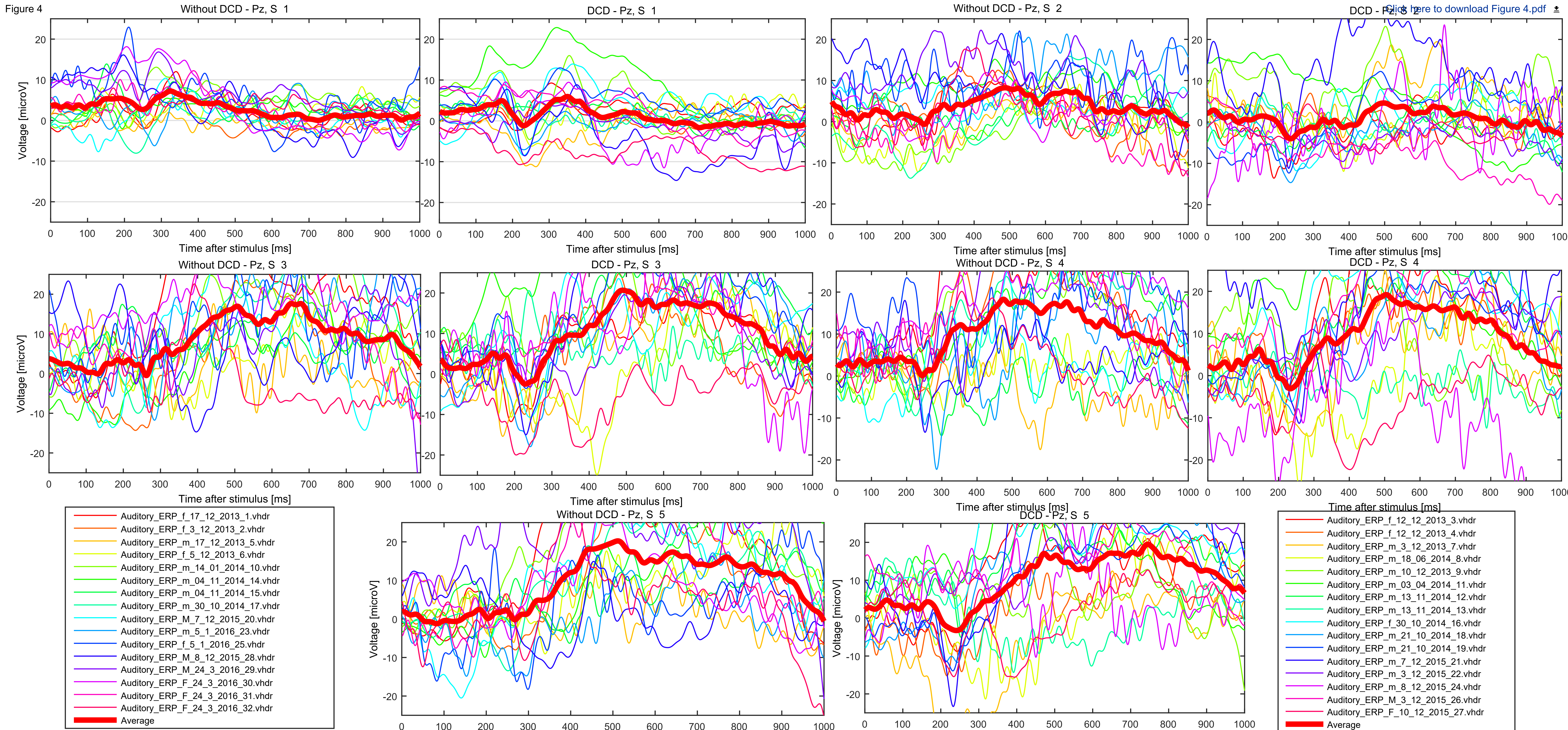

Figure 5 Click here to download Figure 5.pdf

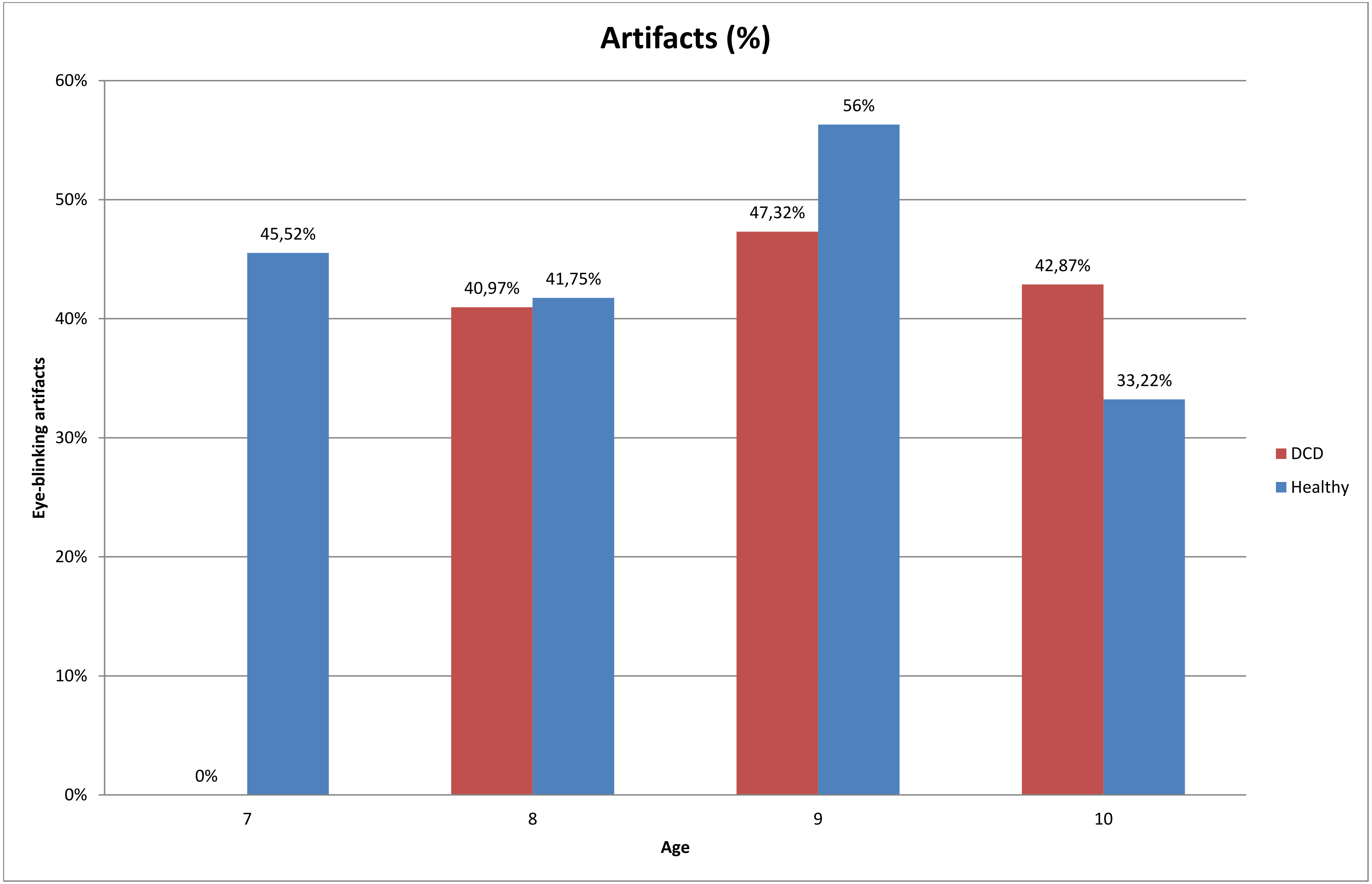

Supplement: GIGA-D-16-00094_Revision_3.pdf [file gix002_GIGA-D-16-00094_Revision_3.pdf]
