# Supplementary material for: Developmental coordination disorder in children – experimental work and data annotation
Source: Gigascience. 2017 Feb 24;6(4):1–6. doi: 10.1093/gigascience/gix002 (PMC5530316; doi:10.1093/gigascience/gix002)
Supplement: Response_to_reviewer_comments_Revision_1.pdf [file gix002_Response_to_reviewer_comments_Revision_1.pdf]

Major Compulsory Revision:

1. Common motor skill assessments that are used in this population, like the MABC-2, BOT-2, or TGMD-2, are relatively fast to administer (~30-45 minutes). Although setting up an EEG cap with 19 electrodes may be very fast, I do not think that the speed alone justifies the use of EEG as a replacement for traditional behavioral assessments. It would be worthwhile to also add the approximate total time (set up + paradigm) and to substantiate the utility of EEG. What information is gained that cannot be gained using behavioral assessments? Moreover, the expense of these behavioral assessments is much reduced compared with most EEG systems (i.e., ~$1000 or less compared with $80,000 for a BrainVision EEG system). As such, the important thing about the use of EEG systems is that they are able to do a better job of discriminating differences or add something unique to our understanding of DCD and the potential comorbidities. These points, the time and cost considerations, could be highlighted in the introduction or discussion.

This discussion was added into the “Theoretical background and purpose of the study” chapter.

2. Given the comorbidity of DCD and other developmental/learning disabilities (e.g., Autism Spectrum Disorder, ADHD, developmental dyslexia), it would be worthwhile to add in the metadata or participant information the existence of comorbidities and how these comorbidities were assessed (e.g., parent report, neuropsychological/educational tests, physician diagnosis). Without this information: A) it would be difficult to use this data repository to reliably combine data from different research groups and B) it would be impossible to use data-driven discrimination procedures to predict group membership (e.g., control, probably DCD, DCD, DCD+ADHD, DCD+ASD, etc.). Based on the introduction, I believe that the ultimate goal would be to address both A and B.

The information about comorbidities have been collected from reports of educational and psychological counselling centres and are now included in the metadata.

3. The total scores from the MABC-2 are not typically reported. The percentiles are more meaningful because the red zone is characterized as at or below the 5th percentile, the amber zone is between the 6th and 15th percentile, and the green is at or above the 15th percentile. As such, the description of the MABC

under 1.2 Participants should be changed to reflect the language in the MABC-2 manual (see above or page 176 of the manual). Also, the table provides “Sum SS” which should be changed to “Total Score”.

We were following the ample system mentioned in:

Henderson, S.E., Sugden, D.D.A.., Barnett, A.L., Corporation, P.: Movement Assessment Battery for Children-2.
London : Harcourt Assessment. Title from Examiner's manual cover (2007).

On page 176 there is a table we used.

We were following the Total test score in the table 3b to obtain two equally sized groups of test subjects. It is definitely simple to switch to percentiles, however, two subjects with the percentile of 16 would fall into the green zone. Now, they are in the amber zone (having the total test score of 63 and 65). Such a strange overlap would mean that with percentiles, we would have one group of 14 and one group of 18 in the situation when getting more subjects is very complicated and nearly impossible.

Minor Compulsory Revisions:
1. In the table, age in months should be used instead of age in years.

The information about ages were updated in the metadata.

2. Were the participants with visual impairments wearing corrective lenses during the task?

The information whether the participants were wearing corrective lenses is mentioned in the metadata.

3. Blink artifacts are easily removed without data loss with algorithms like independent components analysis. These algorithms are now included in EEGlab or BrainVision Analyzer 2 and are very useful for young children and those that tend to blink a lot. Given a reduction in data loss, the paradigm may be able to be

shortened. The authors could provide this as a suggestion.

Independent component analysis is now mentioned in the manuscript. We did not perform ICA because we wanted to provide raw data.

Close
