# Supplementary material for: Developmental coordination disorder in children – experimental work and data annotation
Source: Gigascience. 2017 Feb 24;6(4):1–6. doi: 10.1093/gigascience/gix002 (PMC5530316; doi:10.1093/gigascience/gix002)
Supplement: Response_to_reviewer_comments_Revision_2.pdf [file gix002_Response_to_reviewer_comments_Revision_2.pdf]

The GigaDB citation was added. The "Availability of supporting data" was rewritten as requested.
