# Supplementary material for: Developmental coordination disorder in children – experimental work and data annotation
Source: Gigascience. 2017 Feb 24;6(4):1–6. doi: 10.1093/gigascience/gix002 (PMC5530316; doi:10.1093/gigascience/gix002)
Supplement: Reviewer_2_Report_(Original_Submission).pdf [file gix002_Reviewer_2_Report_(Original_Submission).pdf]

Reviewer Report

Title: Developmental coordination disorder in children - experimental work anddata annotation

Version: Original Submission **Date:** 2/12/2016

Reviewer name: Melissa Pangelinan

Reviewer Comments to Author:

The manuscript is clear and the data collection methods are sound. There is certainly a lack of neuroimaging studies that examine children with DCD and making these data available to the public is important. However, there are several issues that need to be addressed in order for these data to be of use to other researchers (both for the metadata and in the manuscript).

Major Compulsory Revisions

1) I am not sure if traditional results are necessary in a data note submission but future users would greatly benefit from at least one data visualization to determine if these data are of sufficient quality. A figure with the individual ERPs for each group (not the group grand-average ERPs) for each condition would be particularly useful. This would provide users with an idea of the variability of these data, particularly for the children with DCD.

2) 1.1 (page 1): It is not clear why this task is appropriate for understanding the deficits affecting children with DCD. At least one statement regarding deficits in multi-sensory integration and movement time deficits in children with DCD should be included in the purpose statement.

3) 1.2.5 (page 2): Please include a table with participant details. Given the small sample size and the inherent variability of pediatric data, it would be worthwhile to include this information for ALL participants (and not just the children with DCD or suspected DCD). This table should include: age, sex, MABC raw and

percentile scores for the total score and component scores (manual dexterity, ball skills, and balance scores), the degree of hearing impairment, the auditory threshold levels, and whether the child had vision correction.

Minor Essential Revisions

1) 1.2 .1 (page 1): Please include the frequency range of these data (i.e., are any data filters applied to the raw data?).

2) 1.2.4 (page 2): Please include the inter-stimulus interval and jitter in the stimulus presentation.

3) 1.2.4 (page 2): Please include the total testing time for each run.

4) 1.2.5 (page 3): Please state that MABC-2 was used to determine motor abilities and provide the cut-off percentile scores for each group.

5) 1.2.7 (page 3): Please include the test date in the metadata so that the age at testing can be calculated.

6) 1.2.7 (page 3): Any confidential participant information from the metadata (name, email) should be removed. It would be useful to also include the MABC total score or MABC total percentile, level of hearing impairment, and visual correction would be useful to add to the metadata.

7) Figure 1 (page 6): Rather than plotting data with respect to the participant IDs it would be useful to:

a. Plot the individuals by age, since younger children often have more blink artifacts.

b. Color-code the individuals in each group because one would expect the children with DCD to have more eye-blink artifacts.

Level of Interest

Please indicate how interesting you found the manuscript: An article whose findings are important to those with closely related research interests

Quality of Written English

Please indicate the quality of language in the manuscript: Acceptable

Declaration of Competing Interests

Please complete a declaration of competing interests, considering the following questions:

- Have you in the past five years received reimbursements, fees, funding, or salary from an organisation that may in any way gain or lose financially from the publication of this manuscript, either now or in the future?
- Do you hold any stocks or shares in an organisation that may in any way gain or lose financially from the publication of this manuscript, either now or in the future?
- Do you hold or are you currently applying for any patents relating to the content of the manuscript?
- Have you received reimbursements, fees, funding, or salary from an organization that holds or has applied for patents relating to the content of the manuscript?
- Do you have any other financial competing interests?
- Do you have any non-financial competing interests in relation to this paper?

If you can answer no to all of the above, write 'I declare that I have no competing interests' below. If your reply is yes to any, please give details below.

I declare that I have no competing interests.

I agree to the open peer review policy of the journal. I understand that my name will be included on my report to the authors and, if the manuscript is accepted for publication, my named report including any attachments I upload will be posted on the website along with the authors' responses. I agree for my report to be made available under an Open Access Creative Commons CC-BY license (http://creativecommons.org/licenses/by/4.0/). I understand that any comments which I do not wish to be included in my named report can be included as confidential comments to the editors, which will not be published.

I agree to the open peer review policy of the journal
