# Supplementary material for: Developmental coordination disorder in children – experimental work and data annotation
Source: Gigascience. 2017 Feb 24;6(4):1–6. doi: 10.1093/gigascience/gix002 (PMC5530316; doi:10.1093/gigascience/gix002)
Supplement: Reviewer_2_Report_(Revision_1).pdf [file gix002_Reviewer_2_Report_(Revision_1).pdf]

Reviewer Report

Title: Developmental coordination disorder in children - experimental work anddata annotation

Version: Revision 1 **Date:** 8/31/2016

Reviewer name: Melissa Pangelinan

Reviewer Comments to Author:

I appreciate the author's attention to the comments/changes to the manuscript in response to the reviewers. I believe that the clarity of the methods is much improved. There are a few concerns that remain.

Major Compulsory Revision:

1. Common motor skill assessments that are used in this population, like the MABC-2, BOT-2, or TGMD-2, are relatively fast to administer (~30-45 minutes). Although setting up an EEG cap with 19 electrodes may be very fast, I do not think that the speed alone justifies the use of EEG as a replacement for traditional behavioral assessments. It would be worthwhile to also add the approximate total time (set up + paradigm) and to substantiate the utility of EEG. What information is gained that cannot be gained using behavioral assessments? Moreover, the expense of these behavioral assessments is much reduced compared with most EEG systems (i.e., ~$1000 or less compared with $80,000 for a BrainVision EEG system). As such, the important thing about the use of EEG systems is that they are able to do a better job of discriminating differences or add something unique to our understanding of DCD and the potential comorbidities. These points, the time and cost considerations, could be highlighted in the introduction or discussion.

2. Given the comorbidity of DCD and other developmental/learning disabilities (e.g., Autism Spectrum Disorder, ADHD, developmental dyslexia), it would be

worthwhile to add in the metadata or participant information the existence of comorbidities and how these comorbidities were assessed (e.g., parent report, neuropsychological/educational tests, physician diagnosis). Without this information: A) it would be difficult to use this data repository to reliably combine data from different research groups and B) it would be impossible to use data-driven discrimination procedures to predict group membership (e.g., control, probably DCD, DCD, DCD+ADHD, DCD+ASD, etc.). Based on the introduction, I believe that the ultimate goal would be to address both A and B.

3. The total scores from the MABC-2 are not typically reported. The percentiles are more meaningful because the red zone is characterized as at or below the 5th percentile, the amber zone is between the 6th and 15th percentile, and the green is at or above the 15th percentile. As such, the description of the MABC under 1.2 Participants should be changed to reflect the language in the MABC-2 manual (see above or page 176 of the manual). Also, the table provides “Sum SS” which should be changed to “Total Score”.

Minor Compulsory Revisions:

1. In the table, age in months should be used instead of age in years.

2. Were the participants with visual impairments wearing corrective lenses during the task?

3. Blink artifacts are easily removed without data loss with algorithms like independent components analysis. These algorithms are now included in EEGlab or BrainVision Analyzer 2 and are very useful for young children and those that tend to blink a lot. Given a reduction in data loss, the paradigm may be able to be shortened. The authors could provide this as a suggestion.

Level of Interest

Please indicate how interesting you found the manuscript: An article whose findings are important to those with closely related research interests

Quality of Written English

Please indicate the quality of language in the manuscript: Acceptable

Declaration of Competing Interests

Please complete a declaration of competing interests, considering the following questions:

- Have you in the past five years received reimbursements, fees, funding, or salary from an organisation that may in any way gain or lose financially from the publication of this manuscript, either now or in the future?
- Do you hold any stocks or shares in an organisation that may in any way gain or lose financially from the publication of this manuscript, either now or in the future?
- Do you hold or are you currently applying for any patents relating to the content of the manuscript?
- Have you received reimbursements, fees, funding, or salary from an organization that holds or has applied for patents relating to the content of the manuscript?
- Do you have any other financial competing interests?
- Do you have any non-financial competing interests in relation to this paper?

If you can answer no to all of the above, write 'I declare that I have no competing interests' below. If your reply is yes to any, please give details below.

I declare that I have no competing interests

I agree to the open peer review policy of the journal. I understand that my name will be included on my report to the authors and, if the manuscript is accepted for publication, my named report including any attachments I upload will be posted on the website along with the authors' responses. I agree for my report to be made available under an Open Access Creative Commons CC-BY license (http://creativecommons.org/licenses/by/4.0/). I understand that any comments which I do not wish to be included in my named report can be included as confidential comments to the editors, which will not be published.

I agree to the open peer review policy of the journal
